# Deletion of a Golgi protein in *Trypanosoma cruzi* reveals a critical role for Mn^2+^ in protein glycosylation needed for host cell invasion and intracellular replication

**DOI:** 10.1371/journal.ppat.1009399

**Published:** 2021-03-15

**Authors:** Srinivasan Ramakrishnan, Linn M. Unger, Rodrigo P. Baptista, Teresa Cruz-Bustos, Roberto Docampo

**Affiliations:** 1 Center for Tropical and Emerging Global Diseases, University of Georgia, Athens, Georgia, United States of America; 2 Department of Cellular Biology, University of Georgia, Athens, Georgia, United States of America; University of Texas at El Paso, UNITED STATES

## Abstract

*Trypanosoma cruzi* is a protist parasite and the causative agent of American trypanosomiasis or Chagas disease. The parasite life cycle in its mammalian host includes an intracellular stage, and glycosylated proteins play a key role in host-parasite interaction facilitating adhesion, invasion and immune evasion. Here, we report that a Golgi-localized Mn^2+^-Ca^2+^/H^+^ exchanger of *T*. *cruzi* (TcGDT1) is required for efficient protein glycosylation, host cell invasion, and intracellular replication. The Golgi localization was determined by immunofluorescence and electron microscopy assays. TcGDT1 was able to complement the growth defect of *Saccharomyces cerevisiae null* mutants of its ortholog *ScGDT1* but ablation of *TcGDT1* by CRISPR/Cas9 did not affect the growth of the insect stage of the parasite. The defect in protein glycosylation was rescued by Mn^2+^ supplementation to the growth medium, underscoring the importance of this transition metal for Golgi glycosylation of proteins.

## Author summary

*Trypanosoma cruzi* is the etiologic agent of Chagas disease, which is endemic from North to South America and the most important cause of heart disease in Latin America. *T*. *cruzi* can infect most mammalian nucleated cells and its glycoproteins are needed for its adhesion to cells, and for host cell invasion. Efficient glycosylation of proteins in the Golgi complex requires cations as cofactors. In this work, we found that ablation of a Golgi localized cation transporter prevents normal protein glycosylation, host cell invasion, and intracellular replication, and that protein glycosylation can be rescued by Mn^2+^ but not by Ca^2+^, Mg^2+^, or Zn^2+^, revealing the importance of Mn^2+^ for host parasite interaction.

## Introduction

Manganese (Mn^2+^) is found naturally and is the 12^th^ most abundant element and the 5^th^ most abundant metal. In biological systems, Mn^2+^ is an essential element participating in regulation of metabolism [1], protection from free radical damage [2–4] and activation of enzymes [5–7]. Mn^2+^ can activate several enzymes by either binding directly to them or by interacting with an intermediate substrate, such as ATP [8,9]. Enzymes from many different families such as kinases, oxidoreductases, ligases, isomerases, nucleic acid polymerases, sulfotransferases and glycosyl transferases are known to be activated by Mn^2+^ [10]. Activation of these enzymes requires maintenance of optimal Mn^2+^ concentration within the cells. Mn^2+^ homeostasis is achieved by the concerted action of multiple cation transporters that ensure efficient manganese uptake [11–13]. Very little is known about the expression, activity, specificity, and essentiality of such transporters. Owing to the role of Mn^2+^ in key cellular processes, investigating the significance of these cation transporters has biological relevance.

Recently, the importance of Mn^2+^ transport and homeostasis was specifically highlighted by the identification in *Saccharomyces cerevisiae* of the Golgi-localized cation transporter Gcr1-dependent translation factor-1 protein (Gdt1p) [14], which is an ortholog of the mammalian transmembrane protein 165 (TMEM165) [15]. Gdt1p/TMEM165 is a highly conserved protein among eukaryotes that belongs to the *Uncharacterized Protein Family 0016*, *Pfam PF01169* (UPF0016) [14]. Orthologs of Gdt1p/TMEM165 can be identified by the presence of one or two characteristic E-Φ-G-D-(KR)-(ST) consensus motifs. Less than a decade ago, Foulquier et. al demonstrated the involvement of this protein in congenital disorders of glycosylation (CDG) [15]. Subsequently, its involvement in protein glycosylation was proposed in both yeast and humans [16]. But how Gdt1p/TMEM165 participated in the glycosylation process remained unclear. While some researchers suggested that Gdt1p/TMEM165 plays a role in Ca^2+^ transport [14,17,18], others have argued that it transports Mn^2+^ [19]. Recent evidence supports a role for Gdt1p/TMEM165 in maintaining Golgi Mn^2+^ homeostasis [19–21]. It is believed that the Mn^2+^ transported by Gdt1p/TMEM165 is used as a co-factor for Golgi-localized glycosyl transferases [20]. A concerted action between Gdt1p/TMEM165 and Golgi-localized Ca^2+^/Mn^2+^ATPase Pmr1p (in yeast) or secretory pathway Ca^2+^-ATPase (SPCA) (in humans) has been proposed [21]. Together these proteins maintain the Golgi Ca^2+^/Mn^2+^ homeostasis which is necessary for efficient protein glycosylation [19].

*Trypanosoma cruzi*, the etiologic agent of Chagas disease, possesses a *ScGDT1* ortholog (*TcGDT1*) and we investigated here its relevance. Interestingly, *T*. *cruzi* lacks PMR1/SPCA orthologs and other trypanosomatids, like the *Trypanosoma brucei* group, the agents of sleeping sickness, lack GDT1 orthologs.

*T*. *cruzi* has a complex life cycle with replicative stages in both the insect vector and the mammalian host. *T*. *cruzi* replicates as epimastigote stage in the insect vector midgut and differentiates into infective metacyclic trypomastigote in its hindgut. During a blood meal from the mammalian host, these infective metacyclic trypomastigotes are released with the insect feces deposited at the inoculation site from where they invade the host cells and multiply intracellularly as amastigotes. Following multiple rounds of intracellular replication, the amastigotes differentiate into trypomastigotes and are released into the bloodstream of the mammalian host.

*T*. *cruzi* glycoproteins are particularly important during the mammalian life cycle stages of the parasite. Glycosylated proteins play a prominent role in host cell adhesion, infection and immune evasion [22,23]. In this work, we reveal the role of TcGDT1 in parasite protein glycosylation. TcGDT1 contains two characteristic E-Φ-G-D-(KR)-(ST) consensus motifs, localizes to the parasite Golgi complex and is a functional ortholog of yeast Gdt1p for high Ca^2+^ tolerance. We demonstrate that loss of this protein results in defective protein glycosylation in the parasite which can be completely restored by Mn^2+^, but not by Ca^2+^, Mg^2+^, or Zn^2+^ supplementation. Lastly, we show that loss of TcGDT1 reduces the infectivity and intracellular replication of the parasite underscoring the relevance of Mn^2+^ homeostasis for protein glycosylation in the mammalian life cycle stages of the parasite.

## Results

### Sequence analysis

*T*. *cruzi* GDT1 (TcGDT1) is a 248 amino acid protein with an expected size of 26.58 kDa, encoded by a single copy gene (TcCLB.506211.50). The gene is located on chromosome 40 of *T*. *cruzi* CL Brener Esmeraldo-like genome sequence (curated reference strain, TriTrypDB) [24]. When compared to its orthologs in *S*. *cerevisiae* and *H*. *sapiens*, using Protter [25], TcGDT1 appears to be shorter with fewer transmembrane domains and lacking an N-terminal signal peptide (S1A–S1C Fig). GDT1/TMEM165 from other organisms typically contains two E-Φ-G-D-(KR)-(ST) consensus motifs. Multiple sequence alignment using Clustal Omega [26] indicated that two such E-Φ-G-D-(KR)-(ST) consensus motifs can also be detected in TcGDT1 (S1D Fig). Studies in *S*. *cerevisiae* identified that certain acidic and uncharged polar amino acid residues were required for high Ca^2+^ tolerance in yeast [17]. These amino acid residues, critical for cation transport, are conserved in TcGDT1 (S1D Fig) thereby validating the identity of TcGDT1 as an ortholog of Gdt1p and HsTMEM165. A phylogenetic tree (S2 Fig) depicts the conservation of TcGDT1 and other orthologs in the Euglenozoa group and S1D Fig shows its protein sequence comparison to *S*. *cerevisiae* Gdt1p and *H*. *sapiens* TMEM165.

### Expression and localization of *TcGDT1*

**Fig 1:**
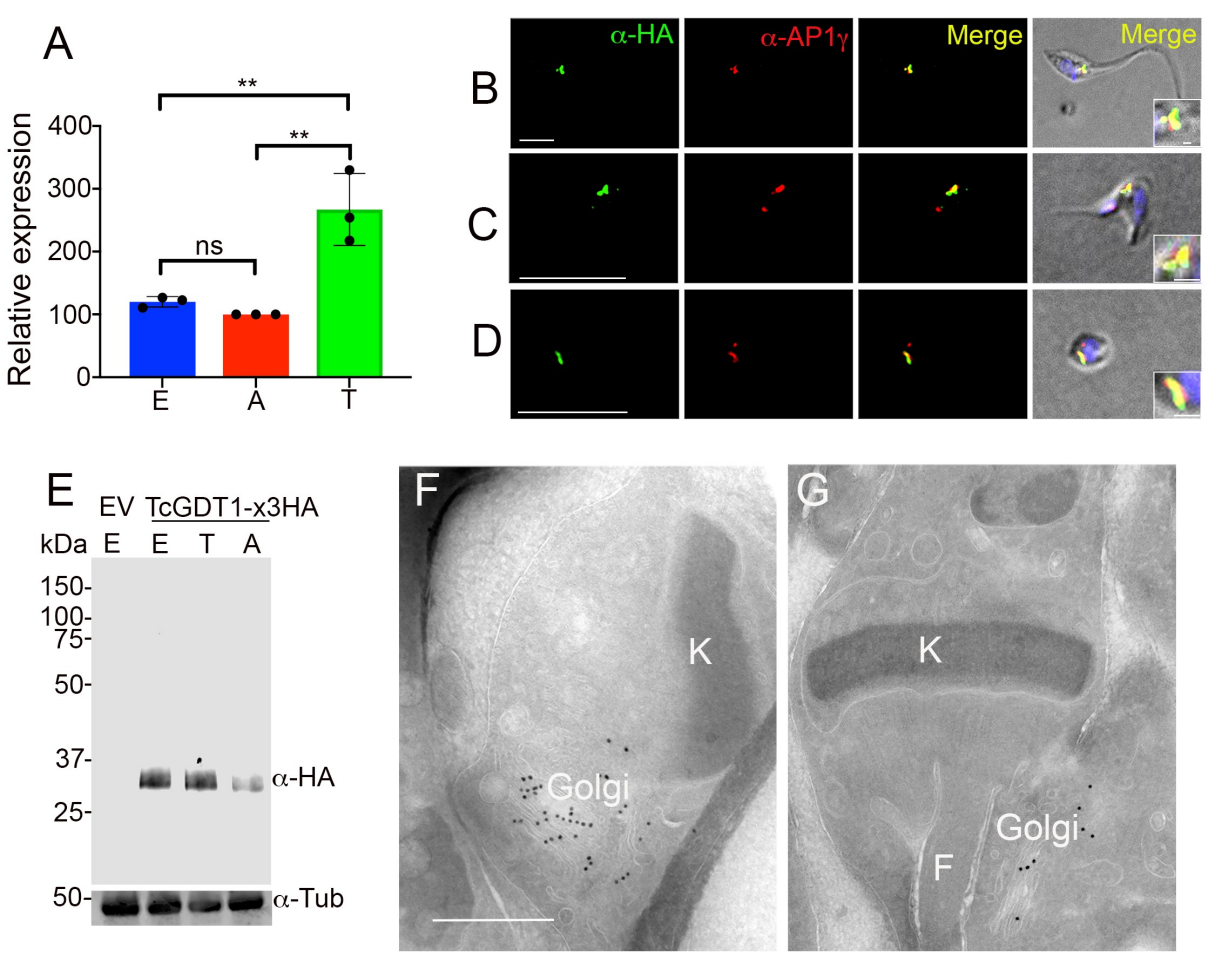
Expression and localization of TcGDT1. (A) Quantitative RT-PCR to determine expression of *TcGDT1* in epimastigotes (E), amastigotes (A) and trypomastigotes (T). Level of expression was normalized to *TcGAPDH* expression. Values are means ± s.d. of n = 3, **p < 0.01, ns = no significant. Statistical analyses were performed using one-way ANOVA and Turkey’s multiple comparisons test. (B-D) Immunofluorescence analysis (IFA) showed co-localization between TcGDT1-3xHA (*green*) and AP1-gamma (Golgi apparatus, *red)* in the merged image (*yellow*) in epimastigotes (Pearsons’ correlation coefficient (PCC) = 0.75 ± 0.05) (B), trypomastigotes (PCC = 0.77 ± 0.008) (C), and amastigotes (PCC = 0.66 ± 0.08) (D). Bars = 5 μm. Insets in differential interference contrast (DIC) images show the colocalization at higher magnification. Bars = 0.5 μm. (E) TcGDT1-3xHA overexpression was confirmed by western blot analysis using anti-HA monoclonal antibodies, using pTREX empty vector (EV) as control cell line. Tubulin (Tub) was used as loading control. (F-G) Cryo-immunogold electron microscopy of epimastigotes overexpressing TcGDT1-3xHA showing localization in the Golgi complex (Golgi). Bar = 0.5 μm. F, flagellum, K, kinetoplast.

First, we sought to determine if *TcGDT1* is expressed in different life cycle stages. We extracted mRNA from three life cycle stages of the parasite and prepared c-DNA for qRT-PCR analyses. When normalized to the expression of *T*. *cruzi* glyceraldehyde 3-phosphate dehydrogenase (*TcGAPDH*), we found that the expression of *TcGDT1* in trypomastigotes is much higher than in epimastigotes or amastigotes (Fig 1A). Both Gdt1p and HsTMEM165 have an N-terminal signal peptide and localize to the Golgi complex. Since the putative TcGDT1 lacks a signal peptide we sought to determine if it could still be targeted to the Golgi complex of the parasite. To test this, we generated a mutant cell line that overexpresses a 3x hemagglutinin (3xHA) tagged version of the protein (TcGDT1-3xHA) by cloning the PCR-amplified open reading frame of the gene into pTREX-n/3xHA plasmid [27]. After selection with G418, resistant cells were analyzed by immunofluorescence (IFA) (Fig 1B–1D) and western blot (Fig 1E) using monoclonal anti-HA antibodies in all three life cycle stages of the parasite. Fluorescence microscopy images showed that TcGDT1 localizes to the Golgi complex, as detected by partial co-localization with the TcAP1γ (Fig 1B–1D), a recently identified marker for Golgi complex in *T*. *cruzi* [28]. This co-localization could be observed in all three life cycle stages of the parasite. To further confirm this localization, we tested the *T*. *cruzi* epimastigotes overexpressing *TcGDT1-3xHA* using electron microscopy (EM) analysis. Representative images (Fig 1F and 1G) clearly demonstrate that TcGDT1-3xHA localizes to the Golgi complex of the parasite. Although, we could not detect an N-terminal signal peptide for TcGDT1, our results from IFA and EM analyses suggest that this protein is targeted to the Golgi complex, consistent with the localization of its orthologs in *S*. *cerevisiae* and *H*. *sapiens*. It is possible that TcGDT1 carries a highly divergent/species specific signal peptide which could not be detected by our bioinformatic analysis. Alternative mechanisms by which a protein could be transported to the Golgi have been reported in other organisms [29].

### Ca^2+^ tolerance of *gdt1*Δ strain expressing *TcGDT1*

**Fig 2:**
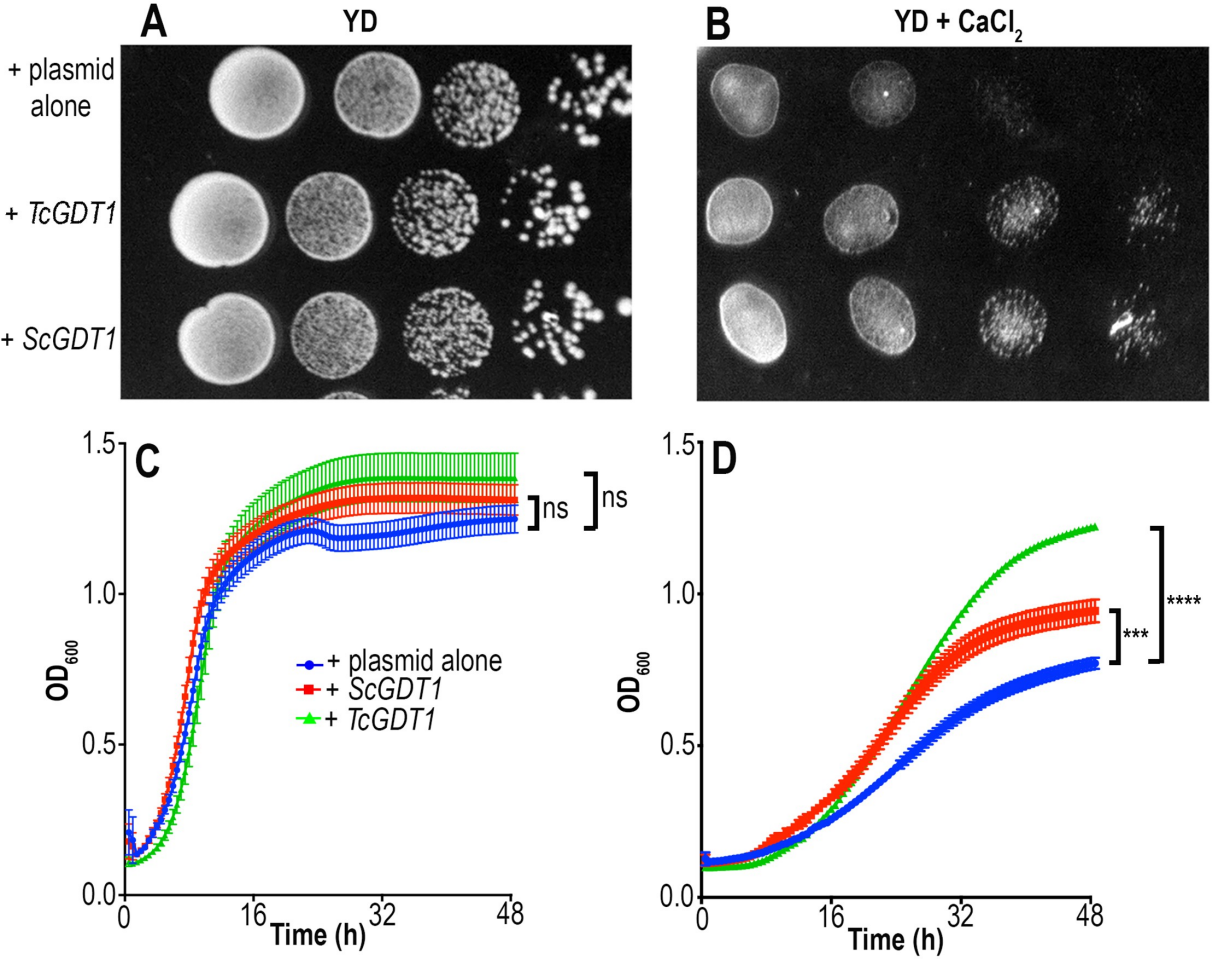
Complementation of yeast *GDT1* mutants. (A, B) Growth of *S*. *cerevisiae GDT1 null* mutant (*gdt1Δ*) transformed with empty plasmid, or complemented with *ScGDT1*, or *TcGDT1* chimera, and precultured in YD medium to an OD_600_ of 0.1. Serial 10-fold dilutions were dropped onto solid YD medium alone (A) or supplemented with 750 mM CaCl_2_ (B). (C-D) Growth of the cultures in liquid YD medium alone (C) or supplemented with 750 mM CaCl_2_ (D) starting at OD_600_ of 0.1 was monitored every 30 min for a total period of 48 hours. Data is representative of three biological replicates, each with five technical replicates. Values are means ± s.d., n = 3; n.s, no significant difference, ***p < 0.005, ****p < 0.001. Statistical analyses were performed using one-way ANOVA and Turkey’s multiple comparisons test.

Loss of Gdt1p affects the growth of *S*. *cerevisiae* under high Ca^2+^ conditions [14]. We tested *gdt1*Δ growth on media containing varying concentrations of CaCl_2_ and found that its growth was inhibited in the presence of 750 mM CaCl_2_ (Fig 2B). Next, we tested whether *TcGDT1* could rescue this growth defect. TcGDT1 contains all the amino acids residues that are critical for Ca^2+^ tolerance in yeast (S1D Fig). Attempts to complement *gdt1*Δ yeast with *TcGDT1* failed, probably because it lacks an N-terminal signal peptide that would be functional in *S*. *cerevisiae* (S1C Fig). Therefore, we synthesized a chimeric *TcGDT1* gene with a sequence encoding the N-terminal signal peptide from Gdt1p and codon optimized it for expression in *S*. *cerevisiae*. We transformed *gdt1*Δ cell line with either empty plasmid, or a plasmid containing either *ScGDT1* or the *TcGDT1* chimera. We tested the growth of these strains on normal YD medium (Fig 2A) or medium containing 750 mM CaCl_2_ (Fig 2B) and found that the growth of the control cell line (*gdt1*Δ with empty vector) was inhibited by CaCl_2_, as expected. This growth defect was rescued by expression of *ScGDT1* in the *gdt1*Δ cell line. Interestingly, this growth defect was also rescued by the chimeric *TcGDT1*. We also tested the growth of these strains in liquid culture (Fig 2C and 2D). We found that the growth of the control cell line (*gdt1*Δ with empty vector) was limited by the presence of 750 mM CaCl_2_, but this defect was rescued by the expression of either *ScGDT1* or *TcGDT1* in the *gdt1*Δ cell line. These results suggested that, similar to ScGDT1 and HsTMEM165, the *T*. *cruzi* GDT1 could also be playing an important role in Golgi cation homeostasis.

### Generation of *TcGDT1* knockout

**Fig 3:**
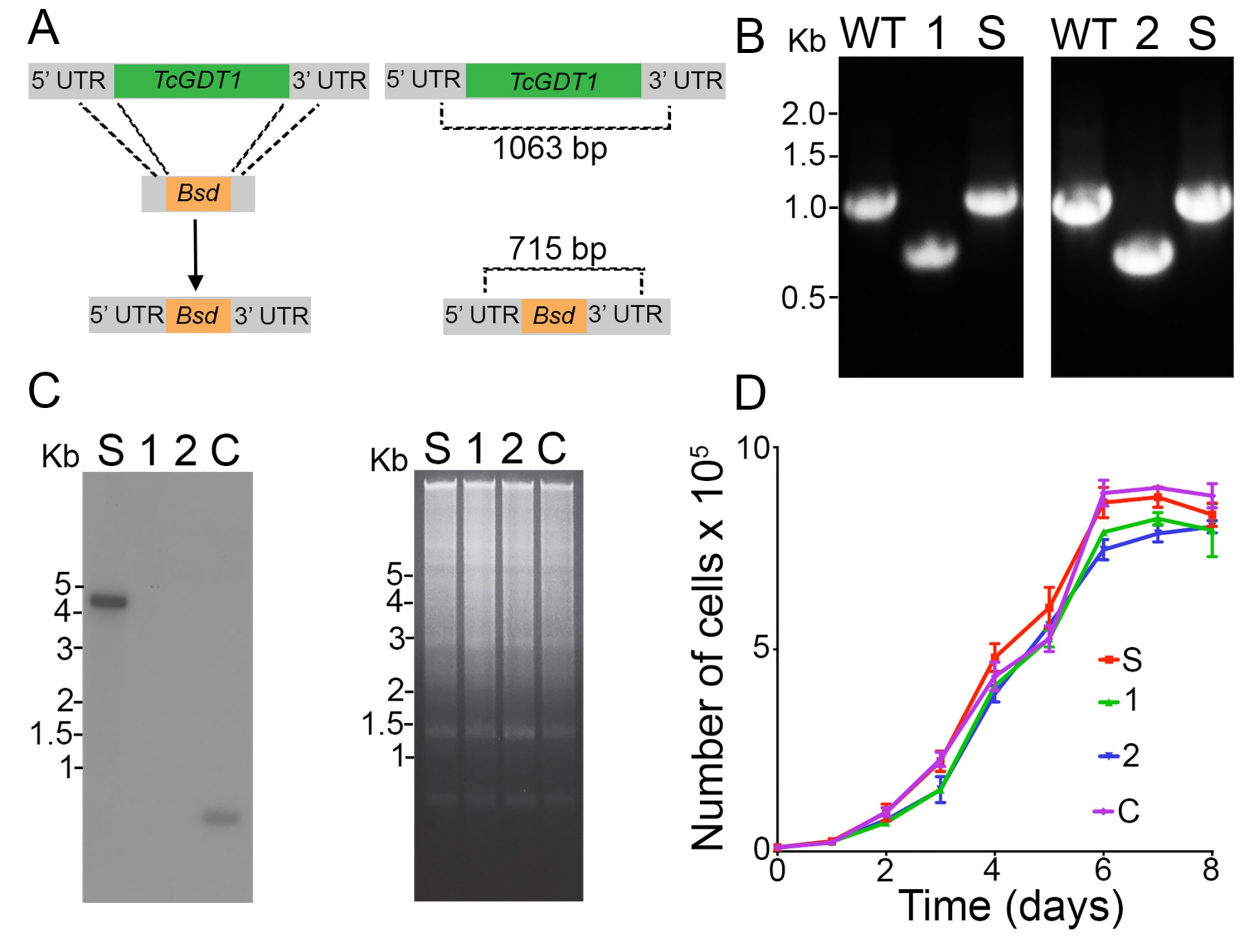
Knockout of *TcGDT1*. (A) Schematic representation of the strategy used to generate a *TcGDT1*-KO cell line by CRISPR/Cas9-mediated genome editing. A double-stranded gDNA break was produced by Cas9 at nt +14 of the *TcGDT1* ORF. DNA was repaired with a blasticidin S-deaminase (*Bsd*) cassette containing 100-bp homologous regions from *TcGDT1* 5’- and 3’- UTRs. Regions amplified by the primers that were used to verify gene replacement by PCR are shown in the right panel. The intact locus generates a PCR product of 1,063 bp, while the replaced locus generates a fragment of 715 bp. (B) PCR analysis showing that *TcGDT1* was ablated at its genomic locus and replaced in genomic DNA of the KO cell line. Lanes: WT, wild type; S, control parasites transfected with a scramble sgRNA; 1 and 2, clone 1 and clone 2 of *TcGDT1*-KO epimastigote. (C) Southern blot analysis of control (S), *TcGDT1-*KO clones 1 and 2 and complemented, C, epimastigote clones. The blot was hybridized with a 227-bp ^32^P labeled probe amplified from *TcGDT1* ORF. The intact locus is detected as a 4 kb band which is missing in the *TcGDT1*-KO clones. A 0.5 kb band can be detected in the complemented clone. Ethidium bromide staining of the Southern blot gel indicating equal loading of DNA in all lanes is shown in the right panel. (D) Epimastigote growth of control (S), *TcGDT1-*KO cl. 1, or *TcGDT1-*KO cl. 2 and complemented *TcGDT1-*KO cl.1. Values are means ± s.d. of n = 3. There were no significant differences in growth by the different clones as compared to the control (S).

To investigate the role of TcGDT1, we generated a *TcGDT1* knockout (*TcGDT1-*KO) cell line using the CRISPR/Cas9 system that has been previously adapted and successfully used in *T*. *cruzi* [30,31]. Using this method, we provided a DNA donor template to promote double strand break repair by homologous recombination and therefore, gene replacement of *TcGDT1* with a resistance marker (the gene encoding blasticidin-S deaminase, *Bsd*) at the genomic locus (Fig 3A) [32]. We generated a molecular construct with a specific sgRNA targeting *TcGDT1* along with constitutive expression of an enhanced Cas9 (eSpCas9) with nuclear localization signal and fused to GFP. This construct was co-transfected with the DNA donor cassette into *T*. *cruzi* epimastigotes. After 5 weeks of selection with G418 and BSD, resistant parasites were obtained. Next, we obtained clonal populations by serial dilutions from this cell line and confirmed the replacement of *TcGDT1* in two clones by PCR using a primer set designed to differentiate between the intact *TcGDT1* locus and the one replaced by *Bsd* (Fig 3B). PCR products were resolved in a 0.8% agarose gel and a band of 1,063 bp was detected in the control cell line transfected with a scrambled sgRNA (S) corresponding to the intact *TcGDT1* locus (Fig 3B). However, for the *TcGDT1*-KO clones, a single band corresponding to the size of the replaced gene (715 bp) was amplified, indicating the generation of a homogeneous knockout population where both *TcGDT1* alleles were deleted. The absence of *TcGDT1* in the *TcGDT1-*KO clonal cell lines was confirmed by Southern blot analysis (Fig 3C). Additionally, we also expressed *TcGDT1-3xHA* in *TcGDT1-*KO clone-1 (cl.1) with a mutated protospacer adjacent motif (*TcGDT1-*KO cl.1 + *TcGDT1-3xHA*_*(ΔPAM*)_) so that this complementing copy is not targeted by the Cas9. The presence of this *TcGDT1-3xHA*_*(ΔPAM)*_ was confirmed by a 0.5 kb band in Southern blot analysis (Fig 3C, C). To determine the biological significance of TcGDT1 we assessed the *in vitro* growth kinetics of *TcGDT1-*KO clones and *TcGDT1-*KO *cl*.*1 + TcGDT1-3xHA*_*(ΔPAM*)_ epimastigotes and compared them with the control (transfected with scrambled sgRNA) cells. We observed that control (scrambled, S) cells and *TcGDT1-*KO epimastigote clones (1, 2) exhibited a similar growth rate (Fig 3D), indicating that this protein is not required for normal proliferation of epimastigotes *in vitro*.

### GP72 glycosylation defect *in TcGDT1-*KO

**Fig 4:**
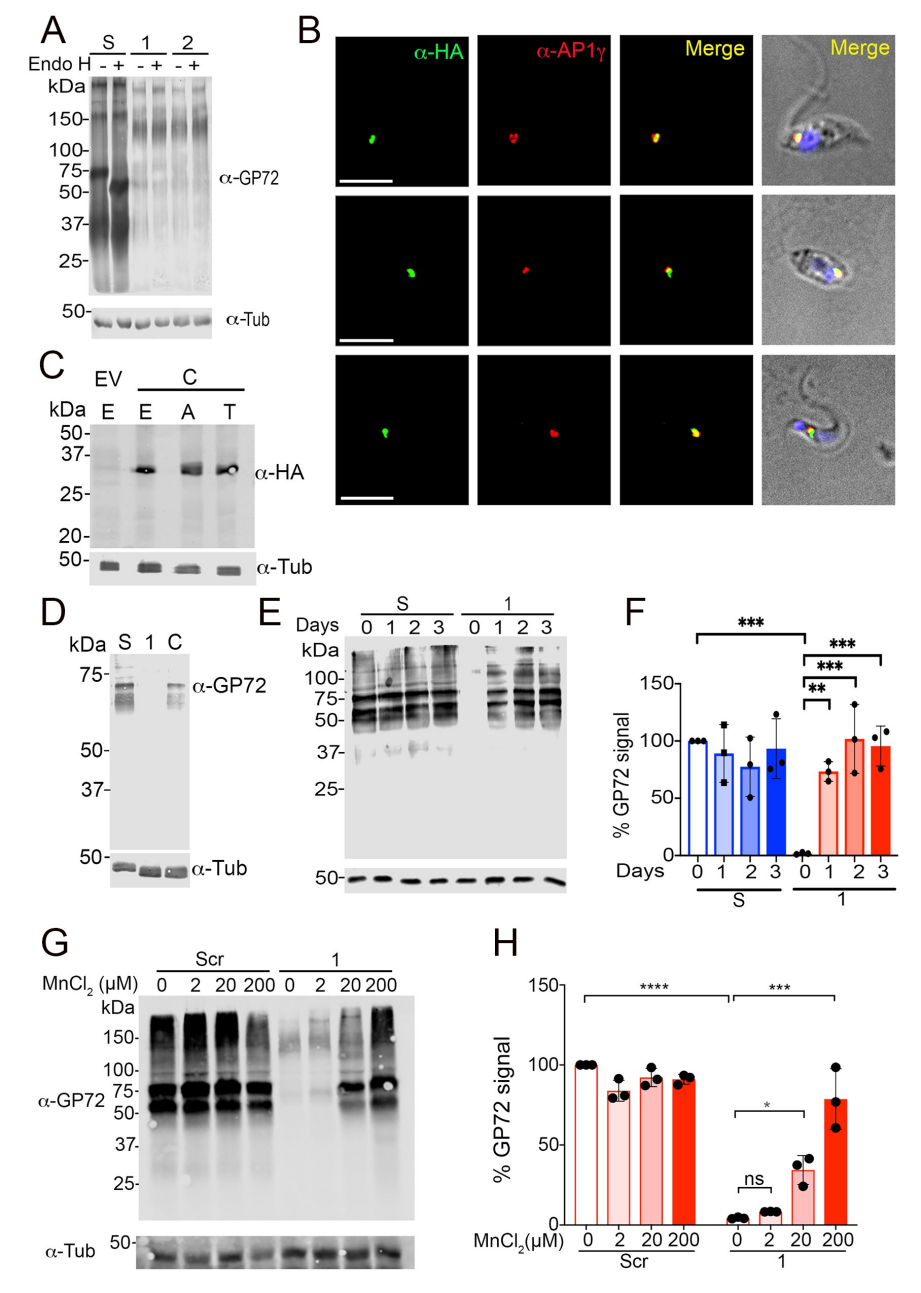
Manganese rescue of protein glycosylation defect. (A) Western blot analysis using WIC29.26 antibody in epimastigotes of control (S) or *TcGDT1-*KO clones 1 and 2 either untreated or treated with endoglycosidase H (endo H). The 72 kDa band corresponding to GP72 is the predominant band detected by WIC29.26 antibody and migrates faster upon endo H treatment indicating that GP72 is a glycosylated protein. The 72 kDa band and the smearing of the control lanes (S) disappear in the *TcGDT1-*KO clones indicating reduced glycosylation. Note that the lane appears different from those shown in E because the gel was run for 1 h and at higher voltage. (B) Immunofluorescence analysis (IFA) shows colocalization of TcGDT1_(ΔPAM)_-3xHA (*green*) and AP1-gamma (*red*) in the merged image (*yellow*) in epimastigotes (*upper panels*), amastigotes (*middle panels*), and trypomastigotes (*lower panels*). Bars = 5 μm. (C) Complementation of *TcGDT1*-KO clone 1 with *TcGDT1*_*(ΔPAM)*_*-3xHA* in epimastigotes [E], amastigotes [A] and trypomastigotes [T] was confirmed by western blot analysis using anti-HA monoclonal antibodies, and pTREX empty vector (EV) as control cell line. Tubulin (Tub) was used as loading control. (D) Western blot analysis using WIC 29.26 antibody in epimastigotes of control (S), *TcGDT1-*KO clone 1, and complemented *TcGDT1-*KO clone 1. The 72 kDa band corresponding to GP72 and the smearing of the lanes of the control (Scr) disappear in the *TcGDT1-*KO clone 1 and are partially restored in the complemented clone. Anti-tubulin was used as loading control. (E) Western blot analysis using WIC 29.26 antibody in epimastigotes of control (S) or *TcGDT1-*KO clone 1 untreated or treated with 20 μM MnCl_2_ for one, two or three days, before protein extraction and SDS-PAGE. The 72 kDa band corresponding to GP72 and the smearing of the lanes of control (S) disappear in the *TcGDT1-*KO clone 1 untreated with MnCl_2_. However, the bands gradually return to the control level by treatment with MnCl_2_ for increasing periods of time. Anti-tubulin (Tub) was used as loading control. (F) The intensity of bands in (E) was quantified by normalizing GP72 signal to the tubulin signal and expressed graphically as a percentage of signal from the control. Values are means ± s.d., n = 3, **p < 0.01; ***p < 0.005. Statistical analyses were performed using one-way ANOVA and Turkey’s multiple comparisons test. (G) Western blot analysis using WIC 29.26 antibody in control (Scr) or *TcGDT1-*KO clone 1 epimastigotes untreated or treated with indicated concentrations of MnCl_2_ for a period of 24 hours before protein extraction and SDS-PAGE. The 72 kDa band corresponding to GP72 and the smearing of the lanes of control (Scr) are greatly reduced in the *TcGDT1-*KO clone 1 untreated with MnCl_2_. However, these bands gradually return to the control level by treatment with increasing concentration of MnCl_2_. Anti-tubulin was used as loading control. (H) The intensity of GP72 bands in (G) was quantified by normalizing GP72 signal to the tubulin signal and expressed graphically as a percentage of signal from the control untreated lane. Values are means ± s.d., n = 3, *p < 0.05, **p < 0.01, ***p < 0.005, ****p < 0.001; ns, not significant. Statistical analyses were performed using one-way ANOVA and Turkey’s multiple comparisons test.

**Fig 5:**
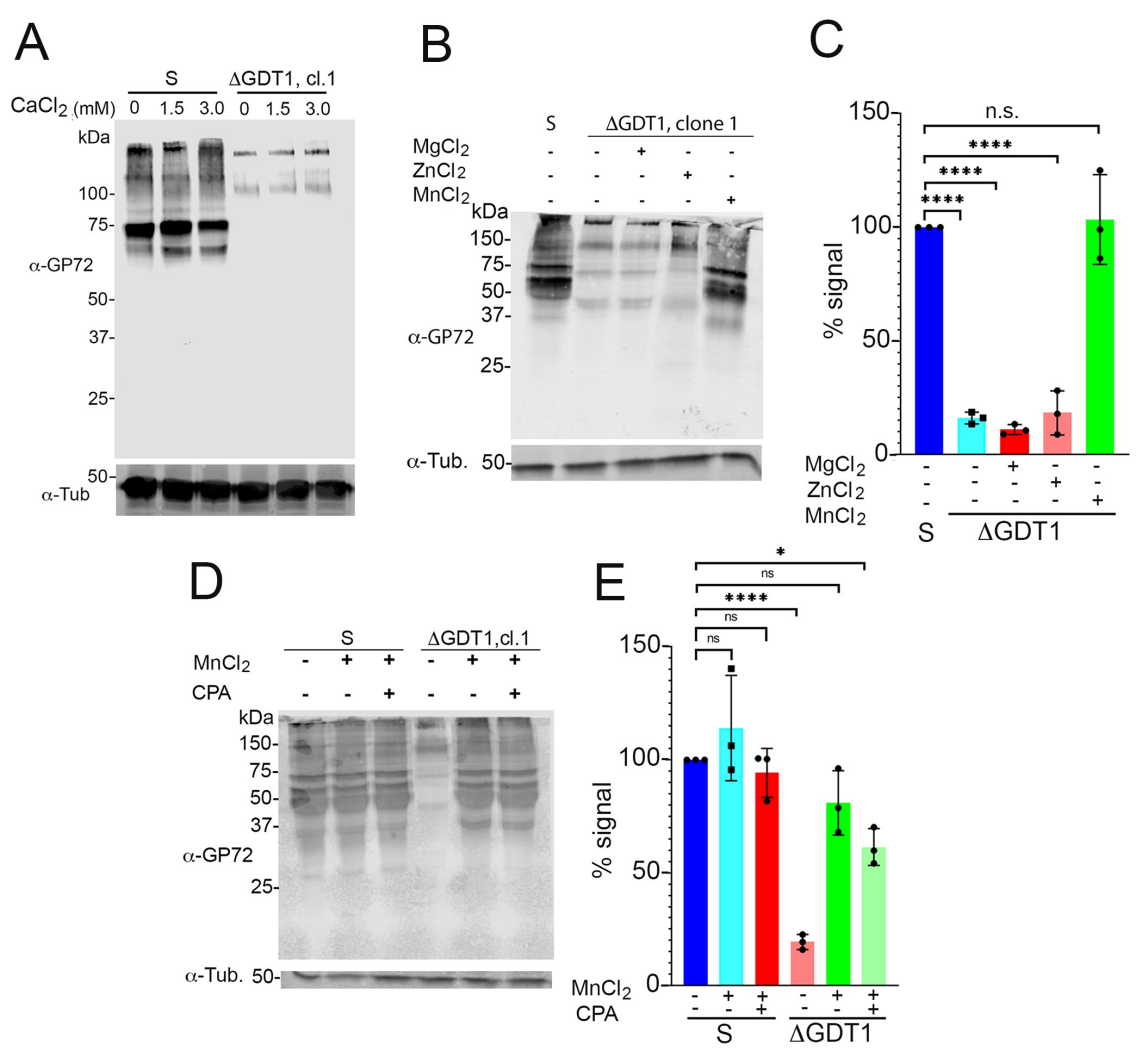
Lack of rescue of glycosylation defect by CaCl_2_, MgCl_2_, and ZnCl_2_ and effect of cyclopiazonic acid. (A) Western blot analysis using WIC 29.26 antibody in epimastigotes of control (S) and *TcGDT1-*KO clone 1, untreated or treated with indicated concentrations of CaCl_2_ for a period of 24 hours before protein extraction and SDS-PAGE. The 72 kDa band corresponding to GP72 and the smearing of the lanes of control (S) disappear or are greatly reduced in the *TcGDT1-*KO clone 1 untreated with CaCl_2_. This band and the smearing in lanes do not return to the control level despite treatment with increasing CaCl_2_ concentrations. Anti-tubulin (Tub) was used as loading control. (B) Western blot analysis using WIC29.26 antibody in epimastigotes of control (S) or *TcGDT1-*KO clones either untreated or treated with 20 μM MgCl_2_, 20 μM ZnCl_2_ or 20 μM MnCl_2_ before protein extraction and SDS-PAGE. The 72 kDa band corresponding to GP72 and the smearing of the lanes of control (S) disappear in the *TcGDT1-*KO clone 1 untreated with MnCl_2_. The bands gradually return to the control level by treatment with MnCl_2_. However, treatment with MgCl_2_ or ZnCl_2_ does not cause the bands to reappear. Anti-tubulin (Tub) was used as loading control. (C) the intensity of bands in (B) was quantified by normalizing GP72 signal to the tubulin signal and expressed graphically as a percentage of signal from the control. Values are means ± s.d., n = 3, **p < 0.01; ***p < 0.005; ****p < 0.0001. Statistical analyses were performed using one-way ANOVA and Turkey’s multiple comparisons test. (D) Western blot analysis using WIC 29.26 antibody in epimastigotes of control (S) or *TcGDT1-*KO clones either untreated or treated with 20 μM MnCl_2_ and 100 μM cyclopiazonic acid before protein extraction and SDS-PAGE. The 72 kDa band corresponding to GP72 and the smearing of the lanes of control (S) disappear in the *TcGDT1-*KO clone 1 untreated with MnCl_2_. The bands gradually return to the control level by treatment with MnCl_2_. Additionally, treatment with CPA does not prevent the bands from reappearing. Anti-tubulin (Tub) was used as loading control. (E) The intensity of bands in (D) was quantified by normalizing GP72 signal to the tubulin signal and expressed graphically as a percentage of signal from the control. Values are means ± s.d., n = 3, *p < 0.05, **p < 0.01; ***p < 0.005; ****p < 0.0001. Statistical analyses were performed using one-way ANOVA and Turkey’s multiple comparisons test.

In *S*. *cerevisiae* and *H*. *sapiens*, Gdt1p and TMEM165, respectively, have been postulated to play a role in Ca^2+^- and Mn^2+^-dependent protein glycosylation [14,17–21]. We therefore performed western blot analyses of lysates from wild type and mutant epimastigotes with WIC 29.26 antibody [33]. WIC 29.26 antibody binds to an epitope of a 72 kDa protein termed GP72 that comprises a phosphosaccharide, containing L-rhamnopyranose, L-fucopyranose, D-galactopyranose, D-galactofuranose, D-xylopyranose, and N-acetylglucosamine [33,34]. As it was reported before [35], western blots of epimastigote lysates of the Y strain, using this antibody, identified a predominant band of 72 kDa, a broad area of weaker signal of lower molecular weight, and weak bands larger than GP72 (Fig 4A). Upon endo H treatment, which cleaves the bond between two N-acetylglucosamine subunits directly proximal to the asparagine residues of glycoproteins and has specific high activity against mannose-rich oligosaccharide modifications [36], this GP72 band migrated faster and resolved at a lower molecular weight confirming GP72 glycosylation in control parasites. Interestingly, in *TcGDT1-*KO clones, the overall signal was reduced and a clear band corresponding to GP72 could not be observed (Fig 4A). This effect was not due to the loss of TcGP72 as the flagella of these cells remained attached to their cell body [35]. To demonstrate that this loss of signal was due to the loss of TcGDT1, we show that expression of *TcGDT1-3xHA*_*(ΔPAM)*_ in *TcGDT1-*KO cl.1, which was confirmed by immunofluorescence (Fig 4B) and western blot (Fig 4C) analyses in different stages of the life cycle, partially restores the TcGP72 band and smearing in the lanes (Fig 4D). This labeling defect with antibody WIC 29.26 could then be a consequence of reduced cation transport and subsequent defective glycosylation of *TcGDT1-*KO clones in the Golgi complex. To test this hypothesis, we treated control and *TcGDT1-*KO clone 1 with varying concentrations of MnCl_2_ by direct addition to parasite growth media. After a 1–3 days treatment, cells were harvested, lysates were prepared, proteins were separated by SDS-PAGE and immunoblotted using WIC 29.26 antibody. We observed that in control parasites the TcGP72 band remains unaffected in absence or presence of MnCl_2_ (Fig 4E and 4F). However, in *TcGDT1-*KO clone 1 cells, a gradual reappearance of the TcGP72 epitope that corresponds to the phosphosaccharide that reacts with the antibody and a gradual reappearance of the smearing in the lanes (Fig 4E) was observed after addition of MnCl_2_ to the medium. Moreover, this gradual reappearance of TcGP72 staining was dose-dependent with increasing concentration of MnCl_2_ in the medium (Fig 4G and 4H). Such reappearance could not be observed by supplementing the medium with CaCl_2_ (Fig 5A), MgCl_2_, or ZnCl_2_ (Fig 5B and 5C). These data strongly suggest that a Mn^2+^ deficiency in *TcGDT1-*KO clones resulted in defective glycosylation of TcGP72 and other glycoproteins in these parasites. It has been described that the rescue of Golgi glycosylation defects in TMEM165-KO HEK293 cells by extracellular Mn^2+^ involves the activity of a thapsigargin- and cyclopiazonic acid-sensitive pump, probably the sarcoplasmic-endoplasmic reticulum Ca^2+^-ATPase (SERCA) pump that could partially localize to the Golgi complex [21]. The *T*. *cruzi* SERCA pump is insensitive to thapsigargin but is cyclopiazonic acid-sensitive [37]. We then preincubated the cells with cyclopiazonic acid, which, however did not inhibit the Mn^2+^ rescue of GP72 glycosylation (Fig 5D and 5E, compare bands 5 and 6).

**Fig 6:**
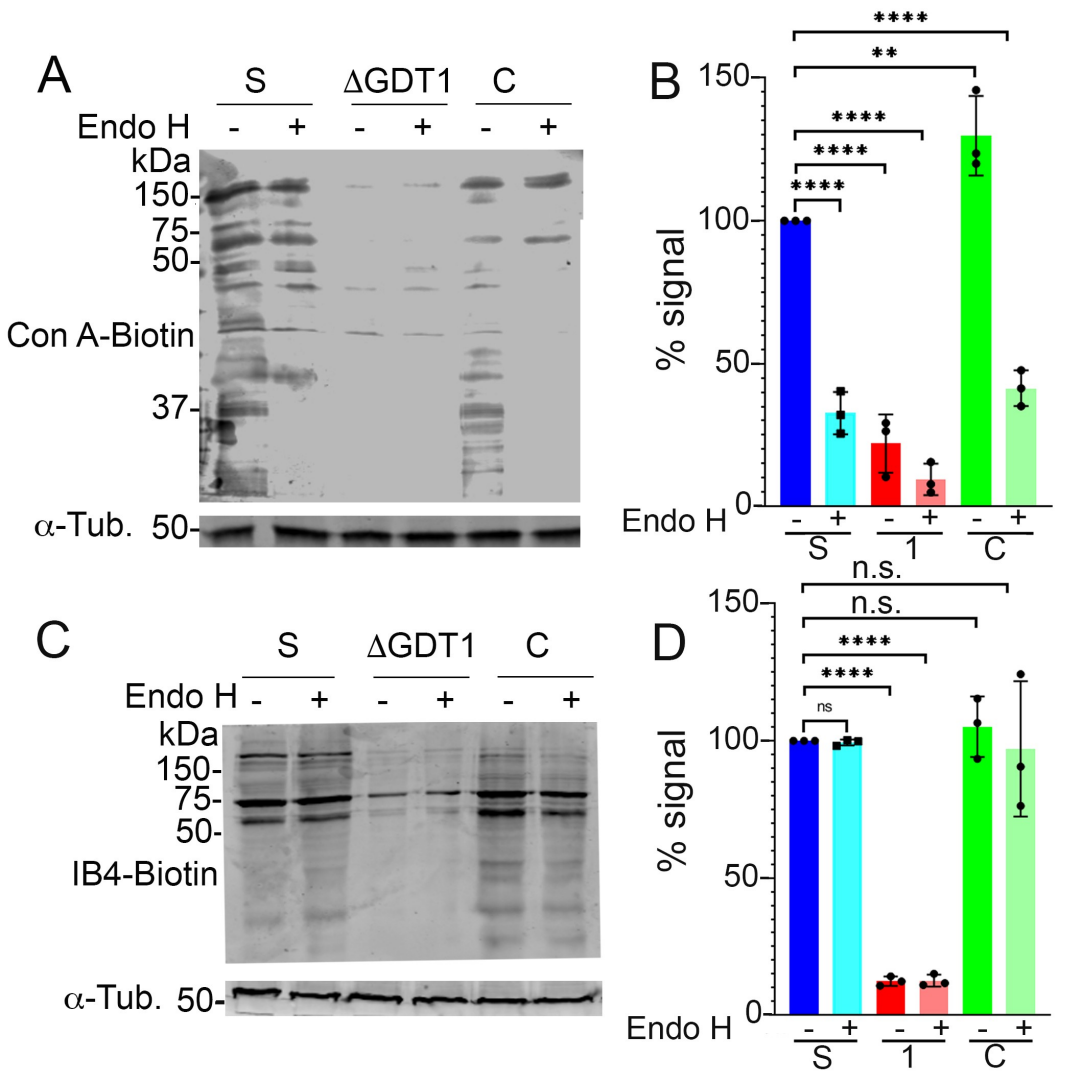
Concanavalin A-biotin, and IB4-biotin staining. (A) Western blot analysis using biotin conjugated concanavalin-A in epimastigotes of control (S) or *TcGDT1-*KO clones either untreated or treated with endoglycosidase H (endo H). Upon ConA labelling, the smearing of the control lanes (S) disappear in the *TcGDT1-*KO clones indicating reduced glycosylation. This smearing is again visible to some extent in the complemented (C) cell line. Anti-tubulin (Tub) was used as loading control. (B) The intensity of bands in (A) was quantified by normalizing ConA signal to the tubulin signal and expressed graphically as a percentage of signal from the control. Values are means ± s.d., n = 3, **p < 0.01; ***p < 0.005; ****p < 0.0001. Statistical analyses were performed using one-way ANOVA and Turkey’s multiple comparisons test. (C) Western blot analysis using biotin conjugated isolectin-B4 in epimastigotes of control (S) or *TcGDT1-*KO clones either untreated or treated with endoglycosidase H (endo H). Upon isolectin-B4 labelling, the smearing of the control lanes (S) disappear in the *TcGDT1-*KO clones indicating reduced glycosylation. This smearing is again visible to some extent in the complemented (C) cell line. Anti-tubulin (Tub) was used as loading control. (D) The intensity of bands in (C) was quantified by normalizing isolectin B4 signal to the tubulin signal and expressed graphically as a percentage of signal from the control. Values are means ± s.d., n = 3, **p < 0.01; ***p < 0.005; ****p < 0.0001. Statistical analyses were performed using one-way ANOVA and Turkey’s multiple comparisons test.

To investigate whether the alterations detected were the result of global glycosylation defects we performed concanavalin A blotting of protein lysates previously treated or not with Endo H. Concanavalin A preferentially binds to mannose residues. We found that this process was affected in *TcGDT1*-KO, clone 1 mutants, and recovered upon gene complementation (Fig 6A and 6B). Endo H treatment reduced the glycosylation of control and mutant lysates (Fig 6A and 6B). We also tested IB4 lectin, which preferentially binds α-D-galactose residues and showed decreased reaction in lysates from mutant parasites without any effect of Endo H (Fig 6C and 6D).

### Differentiation, infection and replication of *TcGDT1-*KO

**Fig 7:**
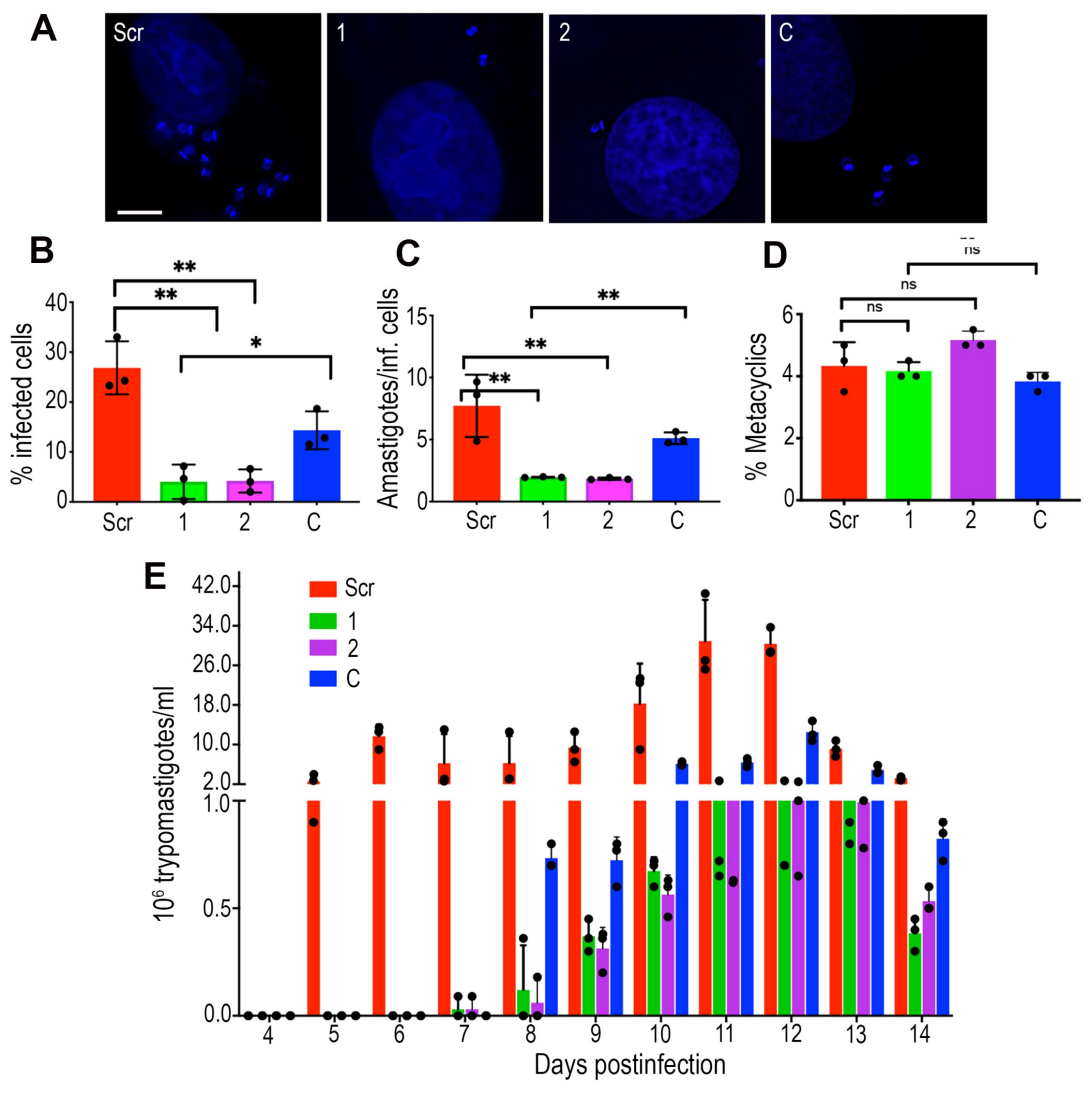
Phenotypic changes in *TcGDT1*-KO. (A) DAPI staining of Vero cells 48 hours after infection with trypomastigotes from control (Scr), *TcGDT1-*KO clones or complemented *TcGDT1-*KO clone 1. (B) Percentage of Vero cells infected by trypomastigotes from control (Scr), *TcGDT1-*KO clones, and complemented *TcGDT1-*KO clone 1. (C) Average number of amastigotes per host cell. (D) Percentage of metacyclic trypomastigotes observed after incubation in TAU 3AAG medium. (E) Total number of trypomastigotes released each day. There was a significant difference in the percentage of infected Vero cells (B) amastigote replication (C), and trypomastigote release from the *TcGDT1-*KO clones (E) when compared to control (Scr). The difference was partially restored upon complementation of *TcGDT1-*KO clone 1. Values are means ± s.d., n = 3, *p < 0.05; **p < 0.01, ***p < 0.005; ns, not significant. Statistical analyses were performed using one-way ANOVA and Turkey’s multiple comparisons test.

Deletion of *TcGDT1* resulted in defective protein glycosylation (Fig 4). Glycosylated proteins or glycoproteins play a central role during the mammalian life cycle stages of the parasite. Therefore, we first investigated the ability of *TcGDT1-*KO epimastigotes to differentiate into metacyclic trypomastigotes under *in vitro* conditions. Metacyclogenesis experiments often resulted in clustering of epimastigotes in the *TcGDT1-*KO cell line, but the control cell line differentiated normally. After multiple attempts, we could perform three biological replicates where parasite clustering was not observed. In these replicates, parasite metacyclogenesis was unaffected upon *TcGDT1* deletion (Fig 7D). We also investigated the ability of *TcGDT1-*KO trypomastigotes to infect tissue-cultured Vero cells. The ability to infect Vero cells was reduced in both *TcGDT1-*KO clones when compared to the control (scrambled) cell line (Fig 7B). This reduction in infection was partially rescued by expressing *TcGDT1* with mutated PAM (Fig 7B). Further, we assessed the replication efficiency of intracellular amastigotes. The ability to replicate within Vero cells was reduced in both *TcGDT1-*KO clones when compared to the control (Scr) cell line (Fig 7A–7C). This deficiency in replication was partially rescued by complementation with an exogenous copy of *TcGDT1* (Fig 7A and 7C). Finally, we enumerated the total number of trypomastigotes released from infected Vero cells on a daily basis. We observed that production of trypomastigotes from both *TcGDT1-*KO clones was significantly delayed starting at about 7 days post infection (compared to 5 days P.I in control cell line) and peaked at around 1 million trypomastigotes/mL of media (compared to 26 million/mL in control cell line) (Fig 7E). This reduction in trypomastigote production by *TcGDT1-*KO clones was partially rescued by complementation of *TcGDT1* with the exogenous copy wherein the trypomastigote production peaked at about 10 million/mL on 12 days post infection (Fig 7E). Together, these data show significant evidence of the role of TcGDT1 in parasite protein glycosylation, infection of host cells, and intracellular replication.

### Loss of Ssp3 epitopes in *TcGDT1-*KO

**Fig 8:**
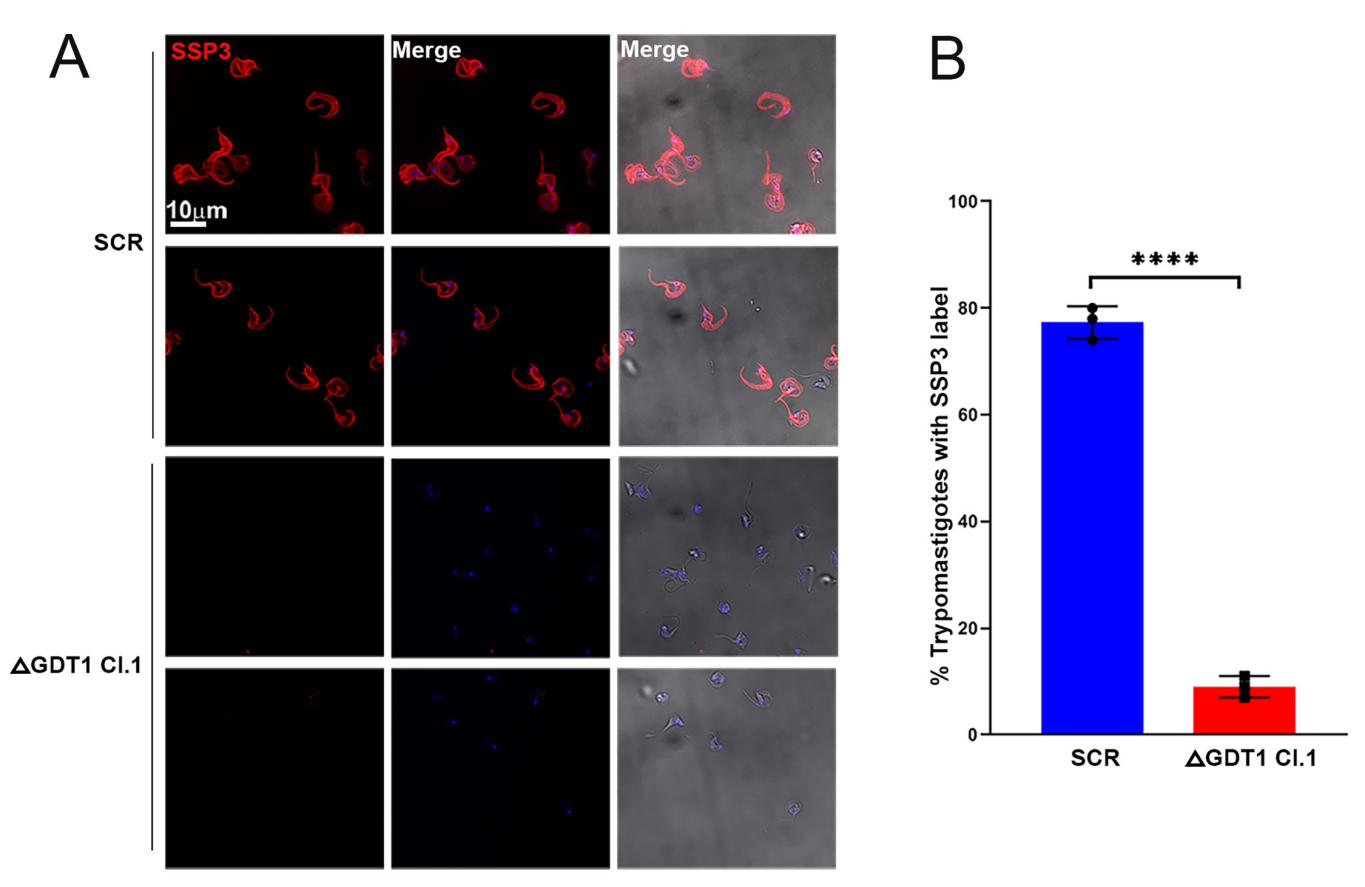
Effects on trypomastigotes Ssp3 expression. (A) Ssp3 expression in control (Scr) and *TcGDT1-*KO clone 1, as determined by immunofluorescence analysis (IFA) using anti-Ssp3 antibodies. Anti-Ssp3 staining of the plasma membrane (*red*) can be observed in control (Scr) but not in *TcGDT1-*KO clone 1 trypomastigotes, using the same antibody dilution and exposure conditions. (B) A total of 200 control (Scr) or *TcGDT1-*KO clone1 trypomastigotes were counted and percent trypomastigotes showing Ssp3 staining in plasma membrane were plotted. Values are means of three biological replicates ± s.d., n = 3, ****p < 0.0001. Statistical analysis was performed using the Student’s *t*-test.

Ssp3 is a trypomastigote-specific epitope recognized by monoclonal antibody 3C9, essentially made by terminal α-2,3-sialic acid residues transferred by a *trans*-sialidase onto O-glycans present in mucin-like proteins [38,39]. Ssp3-bearing molecules, range in size from 60–200 kDa, and participate in the process of trypomastigote internalization [40]. Antibody 3C9 reduces parasite invasion highlighting the importance of the Ssp3 epitope [38,40]. To test whether alteration of this epitope could be involved in the reduced infectivity of trypomastigotes, we used monoclonal antibody 3C9 against Ssp3 [41] in control and *TcGDT1*-KO trypomastigotes. Previously, immunofluorescence assay with anti-Ssp3 antibodies have shown stage specific labeling of *T*. *cruzi* trypomastigotes plasma membrane [42]. Accordingly, we observed that anti-Ssp3 antibodies labeled the plasma membrane of control trypomastigotes (Fig 8A and 8B, Scr). However, in *TcGDT1-*KO trypomastigotes plasma membrane staining by anti-Ssp3 antibodies could not be observed (Fig 8A and 8B, ΔGDT1 Cl.1). These results suggest that altered glycosylation in *TcGDT1-*KO parasites affects the Ssp3 epitope. Such an effect can be expected to affect also other glycoproteins that play a crucial role in *T*. *cruzi* invasion and replication.

## Discussion

Our results provide conclusive evidence regarding the identity and role of TcGDT1. The protein localizes to the Golgi complex of different stages of the parasite, as demonstrated by immunofluorescence and immunoelectron microscopy assays, and can complement the growth defect observed in yeast *gdt1*Δ mutant in the presence of high Ca^2+^, providing functional evidence that it can act as a Ca^2+^/H^+^ exchanger in yeast. The loss of *TcGDT1* does not affect the growth of epimastigotes, but severely reduces the ability of trypomastigotes to invade host cells and replicate as intracellular amastigotes. In addition, protein glycosylation is severely affected in the *TcGDT1 null* mutants. This defect is rescued by Mn^2+^ but not by Ca^2+^, Mg^2+^, or Zn^2+^ supplementation to the growth medium, highlighting the physiological role of TcGDT1 as a Mn^2+^/H^+^ exchanger in the Golgi complex of *T*. *cruzi*.

Bioinformatic analyses revealed that TcGDT1 orthologs are widely distributed in parasitic as well as free-living trypanosomatids, although an ortholog is absent in *T*. *brucei*. TcGDT1 contains two characteristic E-Φ-G-D-(KR)-(ST) consensus motifs. While some bacterial orthologs contain a single E-Φ-G-D-(KR)-(ST) consensus motif, in eukaryotes it is more commonly found as two copies assembled in opposite orientation separated by a central cytosolic loop region. Consistent with this topology, TcGDT1 E-Φ-G-D-(KR)-(ST) consensus motifs also appear to be present in opposing directions with a central cytosolic region. Colinet et al. have identified specific residues in the E-Φ-G-D-(KR)-(ST) consensus motif of Gdt1p that are indispensable for Ca^2+^ tolerance [17]. We found that these residues are conserved in TcGDT1 as well.

Interestingly, TcGDT1 appears to be slightly shorter than its yeast and mammalian counterparts. Both Gdt1p and HsTMEM165 contain an N-terminal signal peptide which localizes the protein to the Golgi complex. Using a prediction tool (SignalP version 5.0) a definitive signal peptide could not be detected at the N-terminus of TcGDT1, and in order to express it in yeast we needed to construct a chimera of the yeast signal peptide and TcGDT1. However, our immunofluorescence and electron microscopy analyses clearly demonstrated localization to the Golgi complex of *T*. *cruzi* for the TcGDT1 protein alone. It is possible that the N-terminal signal peptide in trypanosomatids is evolutionarily different from that in yeast or mammals, thereby making its identification difficult using prediction tools. Previous studies have supported this idea by showing that h-regions of signal peptides from trypanosomatids contain unique species-specific h-motifs [43,44]. Alternate mechanisms of transport to Golgi complex such as N-terminal arginine motifs, transmembrane length variation and self-aggregation have also been reported in other organisms [29]. Interestingly, an N-terminal arginine motif (-RR-) can be detected in TcGDT1.

As occurs with the expression of a truncated form of TMEM165 (lacking its first 55 residues) [14], the sensitivity of yeast *gdt1*Δ mutants to high Ca^2+^ concentration was suppressed by expression of *TcGDT1* chimera, indicating the conservation of function among the members of this family, and suggesting a role of the protein in Ca^2+^ homeostasis in yeast. Notably, the full-length version of TcGDT1, as occurs with the full-length version of TMEM165 [14], was not able to complement the absence of Gdt1p in yeast.

GDT1 acts in concert with a Ca^2+^/Mn^2+^ATPase (Pmr1 in yeast and SPCA in mammals) in maintaining Golgi Ca^2+^ and Mn^2+^ levels [19,45]. However, a Golgi-Pmr1/SPCA ortholog has not been identified in *T*. *cruzi* [46], suggesting the burden of Golgi Ca^2+^/Mn^2+^ homeostasis and parasite protein glycosylation could entirely rest on *T*. *cruzi* GDT1 alone. Absence of a Golgi-Ca^2+^/Mn^2+^ATPase in *T*. *cruzi* also raises a question about regulation of TcGDT1 expression. It has been suggested that the activity of Golgi-Ca^2+^/Mn^2+^ATPase maintains an optimal cytosolic Mn^2+^ concentration in mammalian cells. Maintenance of cytosolic Mn^2+^ concentration by Golgi-Ca^2+^/Mn^2+^ATPase activity is necessary for expression of TMEM165 in mammalian cells [19,21]. Researchers who described Mn^2+^ mediated suppression of *ScGDT1* expression also showed that the first E-Φ-G-D-(KR)-(ST) consensus motif was necessary for this action [19]. This consensus motif is conserved in TcGDT1 suggesting that Mn^2+^ dependent suppression of GDT1 expression may be conserved in *T*. *cruzi* as well.

Deletion of *TcGDT1* from the parasite genomic locus did not result in any growth defect of epimastigotes or in any effect on their differentiation to metacyclic forms. In agreement with the protein glycosylation defects caused by deletion of its orthologs in yeast [47] and humans [19], we observed defective labeling of the carbohydrate epitope of TcGP72 and other proteins in epimastigotes lacking TcGDT1. *TcGDT1-*KO cells did not show flagellar detachment, as occurs with *TcGP72-*KO cells [35], indicating that TcGP72 protein levels were not affected, and that the carbohydrate epitope of TcGDT1 is not necessary for flagellar attachment. The results indicate that the carbohydrate epitope responsible for GP72 labeling is altered in the *TcGDT1*-KO cells, suggesting an alteration in protein glycosylation. This defect could be completely rescued by supplementing parasite growth media with MnCl_2_ but not with CaCl_2,_ MgCl_2_ or ZnCl_2_, supporting the hypothesis [16] that Mn^2+^ transported by GDT1 would be used as a co-factor for Golgi-localized glycosyl transferases. In contrast to the results reported with HEK293 cells [21], cyclopiazonic acid treatment did not prevent Mn^2+^ rescue of the glycosylation defect in *TcGDT1*-KO cells. It has been reported that the Golgi complex of mouse epithelial cells possess a magnesium transporter (MMgT2) that also mediates Mn^2+^ transport when expressed in *Xenopus* oocytes [48]. It is possible that a similar transporter could also be involved in the Mn^2+^ rescue of the glycosylation defect in *T*. *cruzi*.

Glycoproteins play a particularly important role during the mammalian life cycle stages of *T*. *cruzi*. *T*. *cruzi* Complement Regulatory Protein (CRP) is a glycoprotein expressed by trypomastigotes that confers resistance against the lytic activity of the host complement system [23]. Gp82 and Gp35/Gp50, which mediate host cell invasion by activating signaling cascades and Ca^2+^ mobilization, are both GPI anchored glycoproteins [22,49]. The glycoprotein GP90, which is specific for metacyclic trypomastigotes, is responsible for receptor-mediated binding to host-cells during invasion, and the surface glycoprotein Tc85 [50] plays a chief role in host cell invasion by tissue culture derived trypomastigotes [22]. *Trans*-sialidase, a parasite GPI anchored enzyme that transfer sialic acid from host glycoconjugates to the parasite mucin-like molecules has been implicated in cell invasion and escape from the parasitophorous vacuoles (PVs) [38,51]. In this regard, we observed reduced Ssp3 staining in *TcGDT1-*ΚΟ parasites. Ssp3 antibody recognizes an epitope on some *T*. *cruzi* glycoproteins that are required for host cell invasion [40,41]. Reduced Ssp3 staining in *TcGDT-*KO parasites suggested that defective glycosylation due to *TcGDT1* deletion affected expression or proper localization of Ssp3 containing glycoproteins which in turn could affect host cell invasion. In agreement with the relevance of protein glycosylation for *T*. *cruzi* infection, deletion of *TcGDT1* had marked effects on trypomastigotes host cell invasion. Unexpectedly, amastigote replication was also severely affected, and both invasion and replication were rescued by complementation with an exogenous *TcGDT1* gene. These results suggest that TcGDT1 could be playing an important role in host cell invasion and replication by modulating parasite protein glycosylation. Alternatively, the low replication rate and differentiation seen in amastigotes could be due to an overall problem in the homeostasis in cellular Mn^2+^. A recent proteomic comparison identified increased protein glycosylation during the mammalian life cycle stages of the parasite [52]. Together with our results, this would suggest that *T*. *cruzi* protein glycosylation is instrumental in host cell stages of the parasite and that Golgi cation homeostasis by TcGDT1 is playing a pivotal role in regulating this process.

## Materials and methods

### Chemicals and reagents

Blasticidin S HCl, BenchMark prestained protein ladder, BenchMark protein ladder, AlexaFluor-conjugated secondary antibodies were purchased from Life Technologies (Grand Island, NY). IRdye- conjugated secondary antibodies were purchased from LI-COR biosciences (Lincoln, NE). Benzonase nuclease was from Novagen (EMD Millipore, Billerica, MA). Antarctic phosphatase, restriction enzymes and Q5 High-Fidelity DNA Polymerase were from New England Biolabs (Ipswich, MA). Quikchange II site directed mutagenesis kit was from Agilent (Santa Clara, CA). Fluoromount-G was purchased from SouthernBiotech (Birmingham, AL). Anti-HA high affinity rat monoclonal antibody (clone 3F10) was purchased from Roche (Roche Applied Science, Mannheim, Germany). Anti-HA rabbit monoclonal antibody for immunoelectron microscopy was purchased from Sigma (St. Louis, MO). Biotinylated concanavalin A and biotinylated *Griffonia simplicifolia* Lectin I (GSL I) isolectin B4 were purchased from Vector laboratories Inc. (Burlingame, CA). Quick-DNA miniprep plus kit, ZR plasmid miniprep-classic kit, Zymoprep Gel DNA recovery kit and DNA clean and concentrator kit were from Zymo Research (Irvine, CA). GoTaq DNA polymerase and T4 DNA ligase were from Promega (Madison, WI). Antarctic phosphatase, DNA restriction enzymes and Q5 High-Fidelity DNA Polymerase were from New England Biolabs (Ipswich, MA). DNA oligonucleotides were purchased from IDT (Coralville, IA). Custom synthesized DNA sequence was from Genewiz (South Plainfield, NJ). Yeast Nitrogen Base (YNB), complete supplement mixture (CSM), individual amino acids and agarose for culturing *S*. *cerevisiae* were purchased from Sunrise sciences (Knoxville,TN). Precision Plus Protein Dual Color Standards and nitrocellulose membranes were from Bio-Rad (Hercules, CA). BLUelf Prestained Protein Ladder was from FroggaBio (Wheatfield, NY). BCA Protein Assay Kit was from Thermo Fisher Scientific (Waltham, MA). Anti-tubulin monoclonal antibody, puromycin, G418, mammalian cell protease inhibitor cocktail (Sigma P8340), other protease inhibitors, and all other reagents of analytical grade were from Sigma (St. Louis, MO). [α-^32^P]dCTP (3,000Ci/mmol) was from Perkin-Elmer. WIC 29.26 [33] was a kind gift from George Cross (Rockefeller University, NY). Ssp3 and 211.F7 monoclonal antibodies were kind gifts of Norma Andrews (University of Maryland, MD) and Stenio Fragoso (Instituto Carlos Chagas/Fiocruz, Curitiba—PR, Brazil), respectively. pMOTag4H plasmid was a gift from Thomas Seebeck (University of Bern, Bern, Switzerland).

### Cell culture

*T*. *cruzi* Y strain epimastigotes were cultured in liver infusion tryptose (LIT) medium containing 10% heat inactivated fetal bovine serum (FBS) at 28°C [53]. CRISPR/Cas9 mutant cell lines were maintained in medium containing 250 μg/ml G418 and 10 μg/ml blasticidin or 5 μg/ml puromycin. Epimastigotes overexpressing TcGDT1-3xHA were cultured in medium containing 250 μg/ml G418. The growth rate of epimastigotes was determined by counting cells in a Neubauer chamber. Tissue culture cell-derived trypomastigotes were obtained from irradiated Vero cells infected with metacyclic trypomastigotes. *T*. *cruzi* trypomastigotes and amastigotes were collected from the culture medium of infected host cells, using a modification of the method of Schmatz and Murray [54] as described previously [55]. Vero cells were grown in RPMI supplemented with 10% fetal bovine serum and maintained at 37°C under 5% CO_2_.

### Construction of phylogenetic tree

To perform the phylogenetic analysis of the TcGDT1 homologs amongst all orthologs from TcGDT1 in the Euglenozoa phylum and their *S*. *cerevisiae* and *H*. *sapiens* orthologs, all retrieved proteins were aligned using the program MAFFT v.7.4 [56]. The resulted alignment was then submitted to the program Modeltest [57] to predict the best amino acid substitution model to be used in the Maximum likelihood phylogenetic tree reconstruction. The best substitution model obtained was Le-Gascuel (LG) [58] with a discrete gamma distribution with 5 rate categories and 387 invariant sites. After getting the best substitution model, we submitted the alignment to the program PhyML v.3.3 [59] using the predicted substitution model and 1000 bootstrap replicates. The tree was visualized using Figtree 1.4.4 (http://tree.bio.ed.ac.uk/software/figtree/).

### Quantitative real-time PCR

Total RNA was isolated from *T*. *cruzi* epimastigotes, amastigotes and trypomastigotes using the TRI reagent (Sigma) by following the manufacturer’s instructions. The total RNA was treated with DNase I to remove genomic DNA contamination. cDNA synthesis was accomplished using the superscript III first-strand synthesis system (Invitrogen) with 100 ng of total RNA used per reaction. Real-time PCR was done using a CFX96 Touch Real-Time PCR Detection System (Bio-Rad) and set up in white opaque polypropylene wells (LightCycler 480 Multiwell Plates 384) in a final volume of 10 μl per reaction. The primers for gene amplification are listed in S1 Table. Reaction mixtures contained 2 μl of sample DNA (100 ng/μl), 5 μl of a master mix iQ SYBR Green Supermix (Bio-Rad) and 4 μl of nuclease-free water with primers at a final concentration of 300 nM. Activation of polymerase was performed at 95°C for 2 min. PCR cycling conditions included 39 cycles of denaturation at 95°C for 10 s and annealing and extension at 60°C for 30 s. SYBR Green fluorescent emission was measured at the end of the elongation step. Subsequently, a melting curve program was applied with a continuous fluorescent measurement starting at 65°C and ending at 95°C (ramping rate of 0.1°C/s). Expression of *TcGDT1* was determined with primers 17 and 18 and normalized to the housekeeping gene glyceraldehyde 3-phosphate dehydrogenase (GAPDH) using primers 19 and 20 (S1 Table). The samples were quantified according to the %Ct method and all the assays were performed at least three times.

### *TcGDT1-*3xHA overexpression

*TcGDT1* open reading frame (747 nt) was PCR amplified (primers 1 and 2, S1 Table) and cloned into the pTREX-n plasmid [27] by restriction sites XbaI/XhoI and subsequently used to electroporate *T*. *cruzi* epimastigotes. Gene cloning was confirmed by PCR and sequencing. TcGDT1-3xHA overexpression was confirmed by immunofluorescence and western blot analysis using anti-HA antibodies.

### Generation of *TcGDT1-*KO

A single guide RNA sequence to target the putative *TcGDT1* gene was PCR-amplified from the plasmid pUC_sgRNA, as previously described [30]. Protospacer was selected using EuPaGDT (Eukaryotic Pathogen CRISPR guide RNA Design Tool, http://grna.ctegd.uga.edu/) [60]. The *TcGDT1* specific protospacer sequence was included in the forward primer while using a common reverse primer for sgRNA amplification. These primers also contained a BamHI restriction enzyme site for cloning into Cas9/pTREX-n [30] plasmid to generate TcGDT1-sgRNA/Cas9/pTREX-n construct. The sgRNA orientation was confirmed by PCR using the specific TcGDT1-sgRNA forward primer and the HX1 reverse primer [30]. Positive clones that generate a 190-bp PCR fragment were also confirmed by sequencing. A scrambled sgRNA (Scr-sgRNA/Cas9/pTREX-n) was used as control. A DNA donor cassette designed to facilitate homology directed repair and replacement of *TcGDT1* ORF was obtained by PCR using a set of 120 nucleotide long primers (ultramers). In these ultramers, first 100 nucleotides corresponded to the 100 nt before the *TcGDT1* start codon (forward ultramer) and the last 100 nt (reverse ultramer) corresponded to the 100 nt after the *TcGDT1* stop codon. In both ultramers, a 20 nt annealing sequence for *Bsd* gene was included for PCR amplification of the *Bsd* cassette. Circular construct *TcPDP*-sgRNA/Cas9/pTREX-n and linear *Bsd* cassette were used to co-transfect *T*. *cruzi* epimastigotes. After about 5 weeks of selection with 250 μg/ml G418 and 10 μg/ml blasticidin, and cloning by limiting dilution, *TcGDT1* gene replacement was verified by PCR. Primers used to generate *ΔTcGDT1* are listed in S1 Table.

### Generation of TcGDT1-KO + TcGDT1_(ΔPAM)_

Site directed mutagenesis was performed as per manufacturer’s instructions using primers 15 and 16 listed in S1 Table and using pTREX-*TcGDT1*-3xHA-neo plasmid as template. Mutagenesis was confirmed by sequencing.

### Generation of *S*. *cerevisiae* strains, transformation and culture

*Gdt1Δ* yeast strain and pRS416 plasmid containing the TPI1 (triose- phosphate isomerase) constitutive promoter was a kind gift from Dr. Pierre Morsomme (UCLouvain, Belgium). *Gdt1Δ* open reading frame was PCR amplified (primers 5 and 6, S1 Table) and cloned into the pRS416 plasmid using BamHI/XhoI restriction enzyme cut sites. *TcGDT1* open reading frame alone or with the *ScGDT1* N-terminal signal peptide was codon optimized for expression in *S*. *cerevisiae* and was synthesized by Genewiz (South Plainfield, NJ) along with restriction enzyme cut sites. This chimeric *TcGDT1* was cloned into the pRS416 plasmid using BamHI/XhoI restriction enzyme cut sites.

*S*. *cerevisiae* cells were cultured at 30°C in YD medium [2% (wt/vol) yeast extract, 2% (wt/vol) glucose]. Cells transformed with plasmids were precultured in synthetic SD medium [0.7% yeast nitrogen base without amino acids, 2% glucose] supplemented with amino acids depending on the selection markers for plasmid maintenance. Solid media were produced by addition of 2% agar to the mixture. To avoid precipitation, CaCl_2_ was always autoclaved separately from the medium. For the synthetic SD medium containing calcium, yeast nitrogen base was replaced by 0.2% yeast nitrogen base without amino acids and ammonium source and supplemented with 76 mM NH_4_Cl [14]. For transformation, overnight culture of *S*. *cerevisiae* was diluted 1:5 in fresh YD media allowed to shake at 30°C and 200 rpm until the OD_600_ reached 1.5–2.0. Cells were pelleted at 650 x *g*, washed with water and resuspended in 0.1 M lithium acetate and kept on ice. When ready for transformation, cells were pelleted at 5,000 x *g*, resuspended in 33.33% PEG3350, 0.1 M lithium acetate, 0.1 mg salmon sperm DNA and 250 ng of plasmid DNA and vortexed for 1 min. Cells were then incubated at 30°C for 30 min, followed by 42°C in a water bath for 30 min, pelleted at 5,000 x *g*, resuspended in water and plated on selective media. After incubation at 30°C for 3–4 days, colonies were cultured in selective liquid media at 30°C overnight, harvested and analyzed by plasmid isolation, PCR confirmation and sequencing.

*S*. *cerevisiae* cells were precultured overnight in 5 mL of YD medium or in SD medium without uracil for cells carrying a plasmid. The cultures were then adjusted to an OD_600_ of 1 and were spotted onto SD solid medium with or without 750 mM CaCl_2_ along with three 10-fold serial dilutions. The plates were incubated at 30°C for 4–6 days and monitored every day. For liquid growth assay the precultured overnight cultures were adjusted to an OD_600_ of 0.1 in SD liquid medium with or without 750 mM CaCl_2_ and their growth rate was monitored in a 96 well plate using a synergy H1 hybrid multi-mode microplate reader (BioTek) driven by Gen5 software for data collection and analysis.

### *T*. *cruzi* transfections

Electroporations were performed as described previously [27]. Briefly, *T*. *cruzi* Y strain epimastigotes in early exponential phase (1–2 X 10^7^ cells/mL) were washed with sterile PBS, pH 7.4, at room temperature (RT) and transfected in ice-cold CytoMix (120 mM KCl, 0.15 mM CaCl_2_, 10 mM K_2_HPO_4_, 25 mM HEPES, 2 mM EDTA, 5 mM MgCl_2_, pH 7.6) containing 25 μg of plasmid construct and 25 μg of donor DNA in 4-mm electroporation cuvettes with three pulses (1500 volts, 25 μF each pulse) delivered by a Gene Pulser Xcell Electroporation System (Bio-Rad). Stable cell lines were obtained and maintained under drug selection (250 μg/ml G418, 10 μg/ml blasticidin, 5 μg/ml puromycin alone or together depending on the cell line). Transfected epimastigotes were cultured in LIT media supplemented with 20% heat-inactivated FBS until stable and clonal cell lines were established.

### Southern blot analysis

To confirm *TcGDT* gene knockout 3 μg of gDNA from control (transfected with scramble sgRNA), *TcGDT1*-KO and *TcGDT1*-KO *+ TcGDT1*_*(*Δ*PAM)*_ epimastigotes were digested with enzyme PstI and BamHI and resolved on a 0.8% agarose gel. Restriction fragments were transferred to nylon membranes (Zeta-probe membrane, Bio-Rad) and hybridized with a ^32^P-labeled probe corresponding to the first 227 nt of *TcGDT1* ORF that was amplified by PCR using primers 11 and 12 (S1 Table) and labeled using [α-^32^P]dCTP (Perkin-Elmer) with random hexanucleotide primers and the Klenow fragment of DNA polymerase (Prim-A-Gene Labeling System). Following hybridization and post hybridization washes, detection was performed with a phosphorimager screen.

### Western blot analysis

Western blot analyses were performed using standard procedures used in our laboratory [27,61,62]. Briefly, control and mutant epimastigotes were harvested separately, washed twice with PBS (pH 7.4) and resuspended in radioimmunoprecipitation assay buffer (RIPA: 150 mM NaCl, 20 mM Tris-HCl, pH 7.5, 1 mM EDTA, 1% SDS, 0.1% Triton X-100) containing a mammalian cell protease inhibitor mixture (diluted 1:250), 1 mM phenylmethylsulfonyl fluoride, 2.5 mM tosyl phenylalanyl chloromethyl ketone (TPCK), 100 μM *N*-(*trans*-epoxysuccinyl)-L- leucine 4-guanidinobutylamide (E64), and Benzonase Nuclease (25 unit/ml of culture). The cells were then incubated for 1 h on ice and protein concentration was determined by BCA colorimetric protein assay according to the manufacturer’s instructions. Thirty micrograms of protein from each cell lysate were mixed with 4X Laemmli sample buffer (125 mM Tris-HCl, pH 7, 10% (w/v) β-mercaptoethanol, 20% (v/v) glycerol, 4.0% (w/v) SDS, 4.0% (w/v) bromophenol blue) before application to 10% SDS-polyacrylamide gels. Separated proteins were transferred onto nitrocellulose membranes with a Bio-Rad transblot apparatus. Membranes were blocked with 5% nonfat dried skim milk in PBS-T (PBS containing 0.1% v/v Tween 20) for 30 min at RT. Next, blots were incubated for overnight at 4°C with a primary antibody, *i*.*e*. monoclonal anti-HA (1:5000), WIC29.26 (1:2,500) and monoclonal anti-tubulin (1:40,000) or a biotinylated lectin, *i*.*e*. biotinylated concanavalin A (1:500) and biotinylated *Griffonia simplicifolia* Lectin I (GSL I) isolectin B4 (1:500). After three washes with PBS-T at RT for 5 min each, blots were incubated with the secondary antibody (goat anti-mouse IgG or goat anti- rabbit IgG, conjugated to IRdye, diluted 1:10,000). Membranes were washed three times with PBS-T, and Western blot images were obtained with a C-DiGit Blot Scanner (LI-COR Biosciences) and processed and quantified using image studio software. For quantification, intensity from entire lane was measured and normalized to the tubulin band intensity and expressed graphically as a percentage of signal intensity from the control.

### Immunofluorescence analysis

Epimastigotes were washed with PBS (pH 7.4) and fixed with 4% paraformaldehyde in PBS (pH 7.4) for 1 h, at RT. Cells were adhered to poly-L-lysine coated coverslips and then permeabilized with 0.1% Triton X-100 for 5 min. Permeabilized cells were blocked with PBS (pH 7.4) containing 3% BSA, 1% fish gelatin, 50 mM NH_4_Cl, and 5% goat serum overnight at 4°C. Then, cells were incubated with a primary antibody [rat anti-HA-Tag (1:500) or monoclonal mouse anti-AP1γ 211.F7 (1:80)], diluted in 1% BSA in PBS (pH 8.0) for 1 h, at RT. Cells were washed three times with 1% BSA in PBS (pH 8.0), and then incubated for 1 h, at RT in the dark with Alexa Fluor 488 –conjugated goat anti-rat secondary antibody (1:1,000) or Alexa Fluor 546 –conjugated goat anti-mouse secondary antibody (1:1,000). Then, cells were washed and mounted on slides using Fluoromount-G mounting medium containing 5 μg/ml of 4’,6- diamidino-2-phenylindole (DAPI) to stain DNA. Controls were performed as described above but in the absence of a primary antibody. Differential interference contrast and fluorescent optical images were acquired with a 100× objective (1.35 aperture) under non-saturating conditions with a Photometrix CoolSnapHQ charge-coupled device camera driven by DeltaVision software (Applied Precision, Issaquah, WA) and deconvolved for 15 cycles using Softworx deconvolution software.

### Cryo-immunogold electron microscopy

Epimastigotes were washed with PBS (pH 7.4) and fixed with 4% paraformaldehyde and 0.05% glutaraldehyde in PBS (pH 7.4) for 1 h on ice. Samples were processed for cryo-immunoelectron microscopy at the Molecular Microbiology Imaging Facility, Washington University School of Medicine. HA-fused protein localization was detected with a rabbit antibody against HA (Sigma) at a 1:200 dilution for 1 h, at RT and goat anti-rabbit conjugated to 18 nm colloidal gold as a secondary antibody for 1h at RT.

### Metacyclogenesis

We followed the protocol previously described by Bourguignon et al. [63] with minor modifications. Control and mutant mid-log phase epimastigotes were obtained after 4 days in LIT medium and incubated for 2 h in triatome artificial urine (TAU) medium (190 mM NaCl, 17 mM KCl, 2 mM MgCl_2_, 2 mM CaCl_2_, 0.035% sodium bicarbonate, 8 mM phosphate, pH 6.9) at room temperature). Then, parasites were incubated for 96 h in TAU 3AAG medium (TAU medium supplemented with 10 mM L-proline, 50 mM sodium L-glutamate, 2 mM sodium L-aspartate, and 10 mM glucose). Following 96 h incubation, parasites were harvested, washed with PBS and fixed with 4% paraformaldehyde in PBS for 1 h, at RT. Cells were adhered to poly-L-lysine coated coverslips and mounted on slides using Fluoromount-G mounting medium containing 5 μg/ml of 4’,6- diamidino-2-phenylindole (DAPI) to stain DNA. Metacyclic trypomastigotes could be differentiated from epimastigotes based on the position of nucleus and kinetoplast identified by DAPI staining. To increase the number of metacyclic forms to infect Vero cells, the contents of the flask were collected and resuspended in medium containing fresh fetal bovine serum and incubated at 37°C for 20 h. The complement in fresh FBS kills epimastigotes whereas metacyclic trypomastigotes are able to survive.

### *In vitro* infection assay

Gamma-irradiated (2,000 radiation-absorbed doses) Vero cells (4.5 X 10^5^ cells) were plated onto sterile coverslips in a 12-well plate and incubated overnight at 37°C, 5% CO_2_, in RPMI medium plus 10% fresh fetal bovine serum. Tissue culture-derived trypomastigote collections were incubated at 4°C overnight to allow amastigotes to settle and separate from swimming trypomastigotes. Trypomastigotes from the supernatants of these collections were counted and 4.5 X 10^6^ parasites were used to infect each coverslip. At 4 h post infection, coverslips were washed extensively with Hank’s balanced salt solution, followed by PBS (pH 7.4), to remove any extracellular parasites. Coverslips were fixed immediately in 4% paraformaldehyde in PBS (pH 7.4), at 4°C for 30 min. Coverslips were washed once with PBS and mounted onto glass slides in Fluoromount-G containing 15 μg/ml DAPI, which stains host and parasite DNA. Coverslips were viewed on an Olympus BX60 microscope to quantify the number of host cells that contained intracellular parasites and the number of intracellular parasites per host cell in randomly selected fields. Three hundred host cells were counted per sample in three independent experiments. To quantify amastigote replication, the following modifications were used: 4.5 X 10^6^ parasites were used to infect each coverslip, and coverslips were allowed to incubate for 48 h post infection at 37°C, 5% CO_2_, prior to fixation and DAPI staining.

### Enumeration of trypomastigote released

Gamma-irradiated Vero cells (7.5 X 10^6^ cells) were plated onto sterile 75 cm^2^ culture flask and incubated overnight at 37°C, 5% CO_2_, in RPMI medium plus 10% fresh fetal bovine serum. Fifty X 10^6^ trypomastigotes were used to infect each flask. For wild type and control (SCR) trypomastigotes, at 8 h post infection, flasks were washed twice with Hanks’ balanced salt solution, to remove any extracellular parasites and allowed to incubate at 37°C under 5% CO_2_. For mutant (*TcGDT1-*KO) trypomastigotes, 8 h infection was insufficient to produce any trypomastigotes. Therefore, a 72 h infection was performed, flasks were washed twice with Hanks’ balanced salt solution, to remove any extracellular parasites and allowed to incubate at 37°C under 5% CO_2_. The released trypomastigotes were collected twice every day and enumerated using a Neubauer’s chamber.

### Statistical analysis

All values are expressed as means ± s.d. Significant differences between treatments were compared using the tests indicated in the figure legends. Differences were considered statistically significant at *p* < 0.05, and n refers to the number of independent biological experiments performed. All statistical analyses were conducted using GraphPad Prism 6 (GraphPad Software, San Diego, CA).

## Supporting information

S1 Fig Sequence analysis of TcGDT1. (A-C) Topology models of *T*. *cruzi* TcGDT1 (TcCLB.506211.50) (C) and orthologs from *S*. *cerevisiae* (YBR187W) (B) and *H*. *sapiens* (ENSP0000370736) (A). GDT1 sequences were analyzed using Protter. (D) Protein sequence alignment was performed using Clustal Omega. Highlighted amino acids indicate the position of critical residues that were identified as indispensable in a yeast Ca^2+^ tolerance screen and that are conserved in *T*. *cruzi*. Asterisks (*) indicate positions which have a single, fully conserved residue; colons (:) indicate conservation between groups of strongly similar properties (Clustal scoring > 0.5); periods (.) indicate conservation between groups of weakly similar properties (Clustal scoring ≤ 0.5).
(TIF)

S2 Fig Phylogenetic tree. Maximum Likelihood phylogenetic tree of TcCLB.506211.50 among several Euglenozoa species, *S*. *cerevisiae*, and *H*. *sapiens* orthologs. The Le-Gascuel 2008 substitution model with a discrete gamma distribution with 5 rate categories and invariant sites was used for the reconstruction with 1000 bootstrap replicates. Bootstraps above 60% are shown in the figure. NCBI or TriTrypDB accession numbers are as follows: *T*. *theileri* (TM35_000032530); *T*. *grayi* (DQ04_01591030); *T*. *rangeli* (TRSC58_06061); *T*. *rangeli 2* (TRSC58_05538); *T*. *cruzi marinkellei* strain B7 (Tc_MARK_4769); *T*. *cruzi_*TCC (C3747_2g195); *T*. *cruzi* CLB (TcCLB.506211.50); *T*. *cruzi* SYLVIO (TcSYLVIO_006058); *T*. *cruzi* Dm28c 2018 (C4B63_4g430); *T*. *cruzi* Dm28C 2017 (BCY84_18590); *T*. *cruzi* Dm28c 2014 (TCDM_00526); *T*. *cruzi* TCC (C3747_18g290); *T*. *cruzi* CLB_2 (TcCLB.508895.70); *Bodo saltans*_lake (BSAL_69795); *Paratrypanosoma confussum* CUL13 (PCON_0052810); *B*. *ayalai* (Baya_005_0040); *Leptomonas pyrrhocoris* H10 (LpyrH10_04_0330); *Leptomonas seymouri* ATCC 30220 (Lsey_0011_0100); *Crithidia fasciculata* strain CfCl (CFAC1_170009700); *Endotrypanum monterogeli* strain LV88 (EMOLV88_190007900); *Leishmania braziliensis* MHOM/BR/75/M2903 (LBRM2903_190010900); *L*. *braziliensis* MHOM/BR/75/2904 (LbrM.19.0630); *L*. *braziliensis* MHOM/BR/75/2904 (LbrM19.2.000630); *L*. *panamanensis* MHOM/COL/81/L13 (LPAL13_190011200); *L*. *panamanensis* strain MHOM/PA/94/PSC1 (LPMP_190290); *L*. *enrietti* strain LEM3045 (LENLEM3045_190008200); *Leishmania* sp. MAR LEM2494 (LMARLEM2494_190008100); *L*. *tarentolae* ParrotTarll (LtaP19.0280); *L*. *amazonensis* MHOM/BR/71973/M2269 (LAMA_000292600); *L*. *mexicana* MHOM/2001/U1103 (LmxM.19.0310); *L*. *major* strain LV39c5 (LMJLV39_190008200); *L*. *major* strain SD 75.1 (LMJSD75_190008100); *L major* strain Friedlin (LmjF.19.0310); *L*. *tropica* L590 (LTR590_190007800); *L*. *arabica* strain LEM1108 (LARLEM1108_190007400); *L*. *donovani* strain LV9 (LdBPK.19.2.000310); L. donovani CLSL (LdCL_190008100); *L*. *aethiopica* L147 (LAEL147_000278300); *L*. *gerbilli* strain LEM452 (LGELEM452_190007700); *L*. *turanica* strain LEM423 (LTULEM423_190007700); *L*. *infantum* JPCM5 (LINF_190008000); *L*. *donovani* strain LV9 (LdBPK_19310.1); *Homo sapiens*_TMEM165 (ENSP0000370736); *S*. *cerevisiae*_GDT1 (YBR187W); *T*. *cruzi*_YC6 (TcYC6_0114230); T. cruzi_BrA4 (TcBrA4_0085790).
(TIF)

S1 Table Oligonucleotide primers used in this work. (DOCX)

S1 Data Numerical data and summary of statistics underlying all graphs. (XLSX)
