# Supplementary figures and images for: Deletion of a Golgi protein in Trypanosoma cruzi reveals a critical role for Mn2+ in protein glycosylation needed for host cell invasion and intracellular replication
Source: PLoS Pathog. 2021 Mar 15;17(3):e1009399. doi: 10.1371/journal.ppat.1009399 (PMC7993795; doi:10.1371/journal.ppat.1009399)

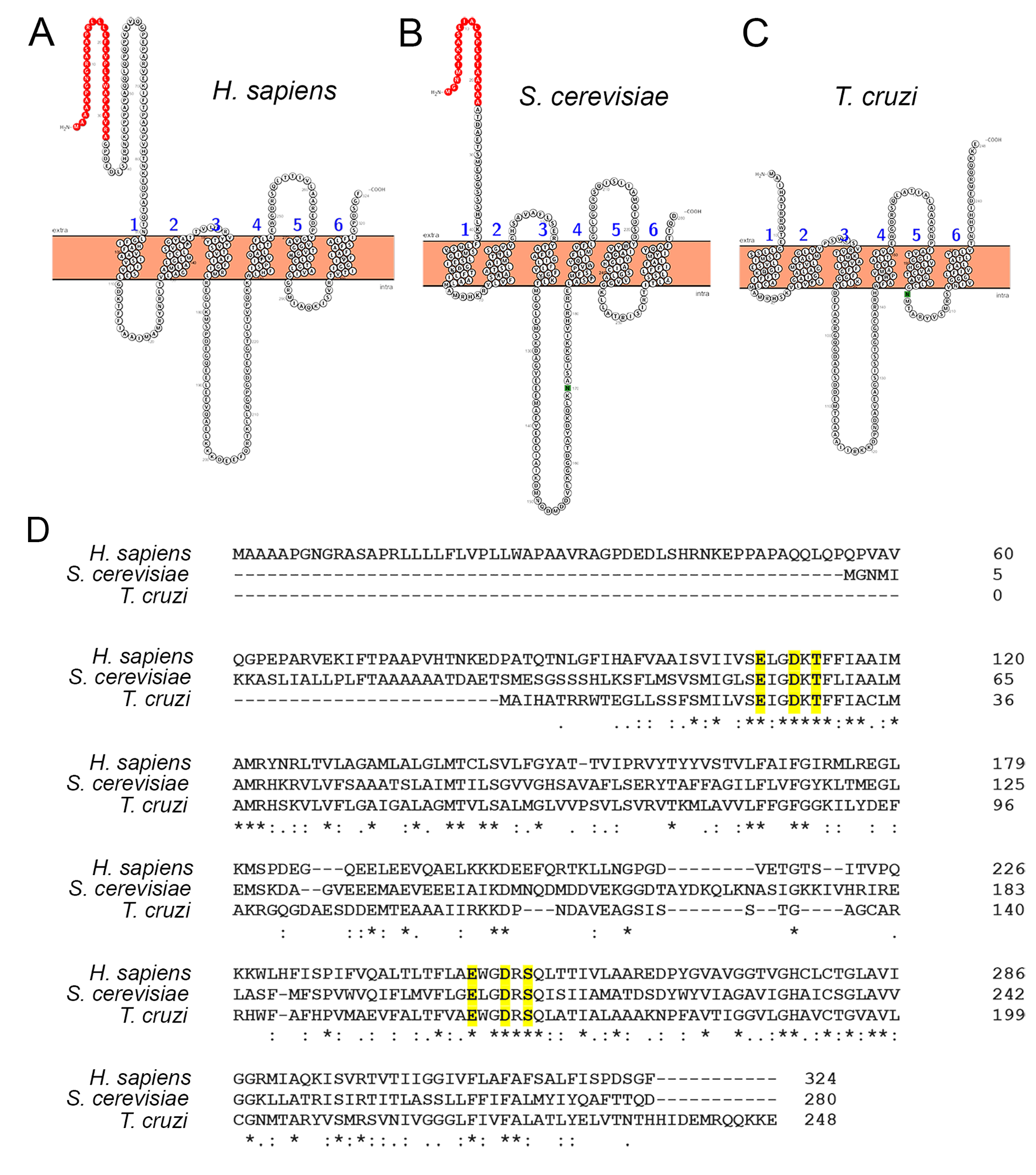

Supplement: S1 Fig — (A-C) Topology models of T. cruzi TcGDT1 (TcCLB.506211.50) (C) and orthologs from S. cerevisiae (YBR187W) (B) and H. sapiens (ENSP0000370736) (A). GDT1 sequences were analyzed using Protter. (D) Protein sequence alignment was performed using Clustal Omega. Highlighted amino acids indicate the position of critical residues that were identified as indispensable in a yeast Ca2+ tolerance screen and that are conserved in T. cruzi. Asterisks (*) indicate positions which have a single, fully conserved residue; colons (:) indicate conservation between groups of strongly similar properties (Clustal scoring > 0.5); periods (.) indicate conservation between groups of weakly similar properties (Clustal scoring ≤ 0.5). (TIF) [file ppat.1009399.s001.tif]

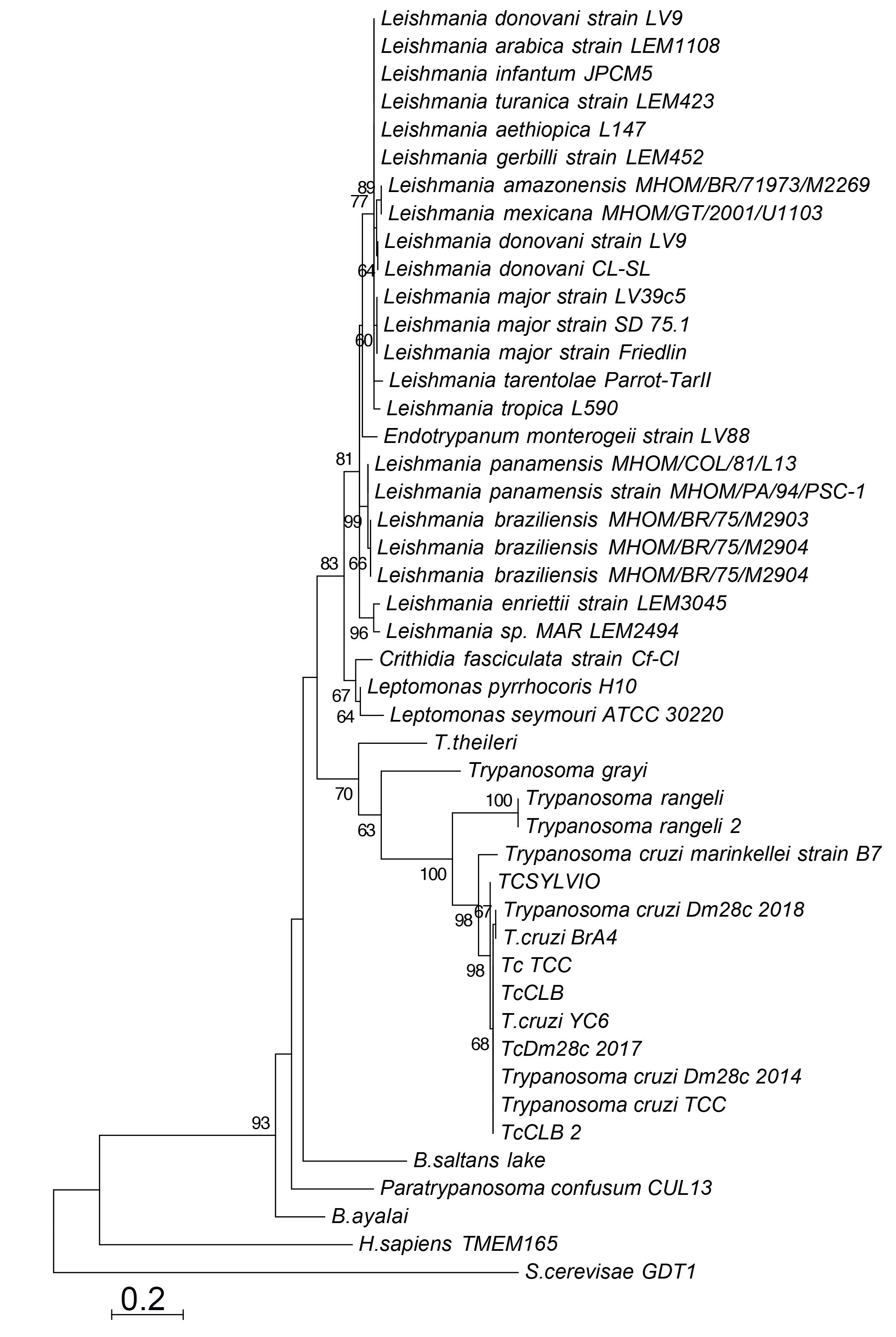

Supplement: S2 Fig — Maximum Likelihood phylogenetic tree of TcCLB.506211.50 among several Euglenozoa species, S. cerevisiae, and H. sapiens orthologs. The Le-Gascuel 2008 substitution model with a discrete gamma distribution with 5 rate categories and invariant sites was used for the reconstruction with 1000 bootstrap replicates. Bootstraps above 60% are shown in the figure. NCBI or TriTrypDB accession numbers are as follows: T. theileri (TM35_000032530); T. grayi (DQ04_01591030); T. rangeli (TRSC58_06061); T. rangeli 2 (TRSC58_05538); T. cruzi marinkellei strain B7 (Tc_MARK_4769); T. cruzi_TCC (C3747_2g195); T. cruzi CLB (TcCLB.506211.50); T. cruzi SYLVIO (TcSYLVIO_006058); T. cruzi Dm28c 2018 (C4B63_4g430); T. cruzi Dm28C 2017 (BCY84_18590); T. cruzi Dm28c 2014 (TCDM_00526); T. cruzi TCC (C3747_18g290); T. cruzi CLB_2 (TcCLB.508895.70); Bodo saltans_lake (BSAL_69795); Paratrypanosoma confussum CUL13 (PCON_0052810); B. ayalai (Baya_005_0040); Leptomonas pyrrhocoris H10 (LpyrH10_04_0330); Leptomonas seymouri ATCC 30220 (Lsey_0011_0100); Crithidia fasciculata strain CfCl (CFAC1_170009700); Endotrypanum monterogeli strain LV88 (EMOLV88_190007900); Leishmania braziliensis MHOM/BR/75/M2903 (LBRM2903_190010900); L. braziliensis MHOM/BR/75/2904 (LbrM.19.0630); L. braziliensis MHOM/BR/75/2904 (LbrM19.2.000630); L. panamanensis MHOM/COL/81/L13 (LPAL13_190011200); L. panamanensis strain MHOM/PA/94/PSC1 (LPMP_190290); L. enrietti strain LEM3045 (LENLEM3045_190008200); Leishmania sp. MAR LEM2494 (LMARLEM2494_190008100); L. tarentolae ParrotTarll (LtaP19.0280); L. amazonensis MHOM/BR/71973/M2269 (LAMA_000292600); L. mexicana MHOM/2001/U1103 (LmxM.19.0310); L. major strain LV39c5 (LMJLV39_190008200); L. major strain SD 75.1 (LMJSD75_190008100); L major strain Friedlin (LmjF.19.0310); L. tropica L590 (LTR590_190007800); L. arabica strain LEM1108 (LARLEM1108_190007400); L. donovani strain LV9 (LdBPK.19.2.000310); L. donovani CLSL (LdCL_190008100); L. aethiopica L147 (LAEL147_000278300); L. gerbilli s [file ppat.1009399.s002.tif]
