# Supplementary material for: Deletion of a Golgi protein in Trypanosoma cruzi reveals a critical role for Mn2+ in protein glycosylation needed for host cell invasion and intracellular replication
Source: PLoS Pathog. 2021 Mar 15;17(3):e1009399. doi: 10.1371/journal.ppat.1009399 (PMC7993795; doi:10.1371/journal.ppat.1009399)
Supplement: S1 Table — (DOCX) [file ppat.1009399.s003.docx]

**S1 Table. Primers used in this work**

| Number | Primer | Sequence |
| --- | --- | --- |
| 1 | Fw_TcGDT1-3xHA_OE | GTACTCTAGAATGGCAATTCACGCCACC |
| 2 | Rv_TcGDT1-3xHA_OE | GTACCTCGAGTTAAGCGTAATCTGGAACATCGTATGGGTAA  GCGTAATCTGGAACATCGTATGGGTAAGCGTAATCTGGAAC  ATCGTATGGGTACTCTTTTTTCTGTTGCCGCATCTCGTC |
| 3 | Fw_TcGDT1_yeast-complementation | GTACAGATCTATGGCAATTCACGCCACC |
| 4 | Rv_TcGDT1_yeast-complementation | GTACCTCGAGCTCTTTTTTCTGTTGCCGCA |
| 5 | Fw_ScGDT1_yeast-complementation | GTACGGATCCATGGGAAATATGATA |
| 6 | Rv_ScGDT1_yeast-complementation | GTACCTCGAGATCCTGAGTTGTGAA |
| 7 | Fw_sgRNA_TcGDT1-KO | GATCGGATCCG**CCATGCGCCACAGTAAGGTTC**GTTTTAG  AGCTAGAAATAGC |
| 8 | Fw_sgRNA_Scrambled | GATCGGATCC**GCACTACCAGAGCTAACTCA**GTT  TTAGA GCTAGAAATAGC |
| 9 | Fw_TcGDT1-KO_ultramer | CAAAGAGGAGAGACAGCCTAAAGAAATAGAAGCCGGGCAT  CACCTCGCCATAAAACAAGTATTGCACATATACTAAGTAAT  TGCGTGAGTTAGAGGCAAAATGGCCAAGCCTTTGTCTCA |
| 10 | Rv_TcGDT1- KO_ultramer | TTTCTTTCTTAATCATCTACTTGTTAGTAATTGCATCAGAACT  CATTAAAAATTGTCCAAAATAAATATTTCCCCCTCCAGCGAC  GTCGCTGTCTGTTTTTTAGCCCTCCCACACATAAC |
| 11 | Fw_TcGDT1-KO_check | TAG AGG CCG ACA CGA GCG A |
| 12 | Rv_TcGDT1-KO_check | CCTTCAGTTCTGCCTCCGAGTA |
| 13 | Fw_TcGDT1_probe | ATGGCAATTCACGCCACCAG |
| 14 | Rv_TcGDT1_probe | GTGACCCGCACCGACAGCAC |
| 15 | Fw_TcGDT1_(ΔPAM)_ | CGCCACAGTAAGGTTCTCGTTTTTCTCGGTGCTAT |
| 16 | Rv_TcGDT1_(ΔPAM)_ | ATAGCACCGAGAAAAACGAGAACCTTACTGTGGCG |
| 17 | Fw_TcGDT1_rtPCR | CGTCTTTGACGTCAAGGCC |
| 18 | Rv_TcGDT1_rtPCR | GCTGAGATGTAGGCAACGAG |
| 19 | Fw_TcGAPDH_rtPCR | GCGAGGTATGTTTCCATGAGAA |
| 20 | Fw_TcGAPDH_rtPCR | TGCCGCATCTCGTCAATG |

In bold, specific protospacer; underlined, restriction enzyme cute site.
